# The co-occurrence patterns and assembly mechanisms of microeukaryotic communities in geothermal ecosystems of the Qinghai-Tibet Plateau

**DOI:** 10.3389/fmicb.2025.1513944

**Published:** 2025-02-04

**Authors:** Bingjie Yan, Xiaodong Li, Nanqian Qiao, Zhen Da, Jiajie Xu, Chuanqi Jiang, Sang Ba

**Affiliations:** ^1^Laboratory of Wetland and Watershed Ecowaters of Tibetan Plateau, Tibet University, Lhasa, China; ^2^Provincial Level of Mitika Wetland Ecosystem Observation and Research Station in Tibet Autonomous Region, Nagqu, China; ^3^Key Laboratory of Aquatic Biodiversity and Conservation, Institute of Hydrobiology, Wuhan, China

**Keywords:** Qinghai-Tibet Plateau, microeukaryotic communities, ecological network stability, geothermal ecosystems, community assembly

## Abstract

Geothermal spring ecosystems, as extreme habitats, exert significant environmental pressure on their microeukaryotic communities. However, existing studies on the stability of microeukaryotic communities in geothermal ecosystems across different habitats and temperature gradients are still limited. In this study, we used high-throughput 18S rDNA sequencing in combination with environmental factor analysis to investigate the co-occurrence patterns, assembly mechanisms, and responses to environmental changes of microeukaryotic communities in sediment and water samples from 36 geothermal springs across different temperature gradients in southern Tibet. The results show that with increasing temperature, the network stability of microeukaryotic communities in sediments significantly improved, while the stability in water communities decreased. The assembly mechanisms of microeukaryotic communities in both sediment and water were primarily driven by undominant processes within stochastic processes. Latitude and longitude were the key factors influencing changes in sediment community composition, while water temperature and electrical conductivity were the major environmental factors affecting water community composition. Additionally, the stability of the geothermal community network was closely related to its response to external disturbances: sediment communities, being in relatively stable environments, demonstrated higher resistance to disturbances, whereas water communities, influenced by environmental changes such as water flow and precipitation, exhibited greater dynamic variability. These findings not only enhance our understanding of the ecological adaptability of microeukaryotic communities in geothermal springs but also provide valuable insights into how microorganisms in extreme environments respond to external disturbances. This is especially significant for understanding how microeukaryotic communities maintain ecological stability under highly dynamic and stressful environmental conditions.

## 1 Introduction

The hot spring ecosystem is typically situated around geothermal activity caused by underground magma and represents a fragile ecosystem akin to an island (Nielson et al., 2019). These hot spring ecosystems enriched with minerals and trace elements, serve as habitats for various microorganisms (Langner et al., 2001). In the medical field, hot springs have a promoting effect on the treatment of conditions such as gastritis, rheumatism, and musculoskeletal disorders (Wangchuk et al., 2021). In terrestrial geothermal hot springs, the main components comprise water and sediment. Hot spring water and sediment, as distinct habitats, result in differences in community diversity, species composition, and community assembly processes (Nevers et al., 2020). Microorganisms are among the oldest life forms on Earth, possessing strong adaptability and the ability to survive in various extreme environments, including extremely low or high temperatures (Miroshnichenko and Bonch-Osmolovskaya, 2006). Hot springs are considered excellent model ecosystems for studying the origins of life, as their chemical conditions are believed to resemble those of early Earth (Woese et al., 1990; Olsen et al., 1994; Li et al., 2015).

Southern Tibet, part of the Qinghai-Tibet Plateau, hosts numerous hot spring ecosystems, making it one of the most geothermally active regions globally. It lies within the Himalayan Geothermal Belt (HGB), which stretches over 3,000 km from Pamir through Tibet to Yunnan and includes more than 600 hot spring systems (Wang et al., 2013; Hochstein and Regenauer-Lieb, 1998; Hu et al., 2022). Although this region boasts rich biodiversity, the high altitude of the Qinghai-Tibet Plateau and strong ultraviolet radiation make its ecosystems highly fragile and extremely sensitive to human disturbances (Ren et al., 2016; Zheng and Zhao, 2017). Therefore, this study focuses on the unique geothermal hot springs of the Qinghai-Tibet Plateau. The microeukaryotic communities in geothermal systems are highly sensitive to climate change, environmental fluctuations, and anthropogenic disturbances (Farrell et al., 2020; Rillig et al., 2019). Moreover, microorganisms within these communities do not exist in isolation; rather, they are intricately interconnected through complex ecological interaction networks (Faust and Raes, 2012; Trivedi et al., 2020). Currently, network analysis has emerged as a crucial tool for assessing microeukaryotic interactions and is widely applied in the study and analysis of various ecosystems (Yang et al., 2023; Zhang et al., 2022). It uncovers the complex interactions among organisms within ecosystems (Clauset et al., 2008; Jeroen and Peer, 2008), enhancing our understanding of community stability and their responses to natural and anthropogenic disturbances (Barberán et al., 2012). By analyzing features such as node attributes, average path length, and average clustering coefficient, we can more accurately reconstruct ecological relationships (Guseva et al., 2022). Network topology features such as modularity and cohesion further allow us to evaluate the robustness of biological communities (Hernandez et al., 2021).

Theories such as niche theory and neutral theory form the foundation for explaining the processes of community assembly. Niche theory suggests that species distribution is determined by both abiotic factors (such as temperature and humidity) and biotic factors (such as competition and symbiosis), while neutral theory emphasizes the role of random processes in shaping community structure (Fargione et al., 2003; Bahram et al., 2016). Conversely, neutral ecology theory posits that the relative abundance and distribution of species are shaped by random processes (such as reproduction, mortality, genetic drift, migration and mutation) rather than biological factors (such as competition), indicating that community assembly is a stochastic process (Zhou and Ning, 2017; Chase and Myers, 2011). However, the formation of microeukaryotic communities is typically influenced by a blend of deterministic and stochastic influences (Berdjeb et al., 2018; Wu and Huang, 2019).

Although high-throughput sequencing technology has been widely applied in global geothermal ecosystem research, such as in Yellowstone National Park in the United States (Meyer-Dombard et al., 2005), Russia (Kublanov et al., 2008), and China (Hou et al., 2013; Wang et al., 2013), studies on geothermal ecosystems in southern Tibet, China, remain insufficient. Previous research has primarily focused on bacterial and archaeal communities (Song et al., 2010; Li and Ma, 2020) and temperature-related changes in microbial community structures. Even among studies on hot springs in southern Tibet, much of the attention has centered on protists (Zhang et al., 2023). However, studies specifically on microeukaryotic communities in southern Tibet’s hot springs are scarce, especially regarding how different habitats respond to temperature gradients. Although some studies have examined the influence of temperature gradients on microbial communities, most categorize temperature simply into low and high ranges without conducting more detailed analyses of temperature variations (Wang et al., 2023). As such, research on the assembly and stability mechanisms of microeukaryotic communities in southern Tibet’s geothermal hot springs remains insufficient and warrants further exploration. However, this study also has some limitations. The impact of external disturbances, such as human activity and climate fluctuations, may influence community composition, introducing variability in the data that is difficult to control.

Against this backdrop, our study seeks to for the first time systematically explore the structure, phylogenetic patterns, co-occurrence networks, and assembly mechanisms of microeukaryotic communities in sediment and water samples from southern Tibet’s hot springs. Using high-throughput sequencing combined with environmental and network analyses, we aim to address three key research questions: (1) How do microeukaryotic community diversity and structure differ between sediment and water? (2) What are the co-occurrence patterns within these communities? (3) Are the assembly mechanisms in these habitats primarily deterministic or stochastic? By addressing these scientific inquiries, our study aims to uncover the driving factors and interaction patterns within the microbial communities of hot springs. This research provides new insights into the stability mechanisms of this unique ecosystem, revealing its resilience to external disturbances. Understanding the assembly mechanisms of microeukaryotic communities in sediment and water helps explore the restoration mechanisms of these ecosystems.

## 2 Materials and methods

### 2.1 Study location and sample collection

**Figure 1:**
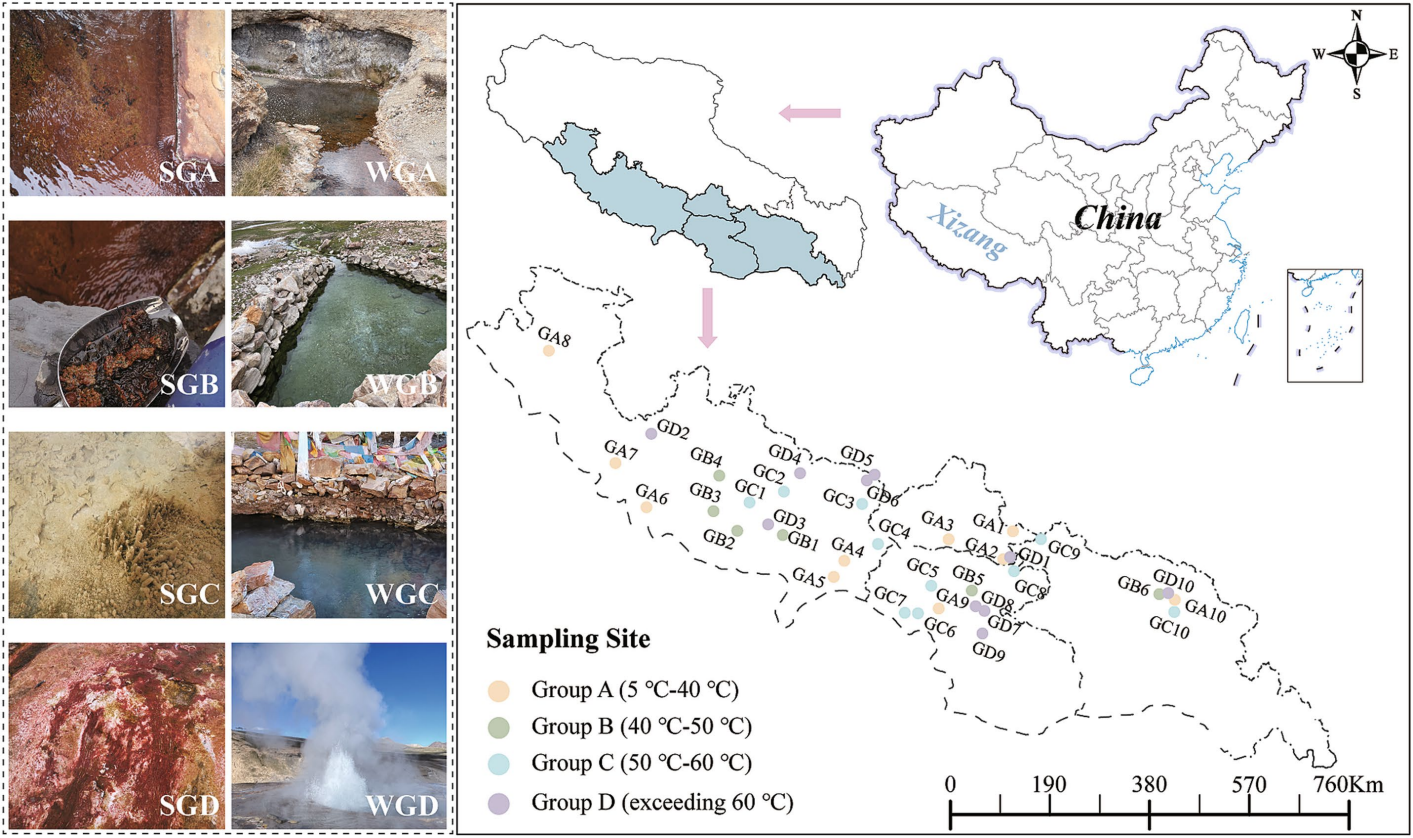
Sampling points distribution. (SGA, Sediment Group A; SGB, Sediment Group B; SGC, Sediment Group C; SGD, Sediment Group D; WGA, Water Group A; WGB, Water Group B; WGC, Water Group C; WGD,Water Group D.) *Statement. Based on the standard map supervised by the Ministry of Natural Resources of the People’s Republic of China [No. GS (2019) 1673] and [No. ZS (2023) 004] retrieved from: http://bzdt.ch.mnr.gov.cn/browse.html?picId=%25224o28b0625501ad13015501ad2bfc0288%2522; http://zrzyt.xizang.gov.cn/fw/zyxz/202004/t20200430_139102.html.

The research was carried out in June and July 2022 in Southern Tibet region (longitude between 83° 36′ E to 95° 3′ E, latitude between 27° 99’ N to 30° 9’ N, elevations ranging from 1972 to 5,027 meters). Surface sediment and water samples were gathered from 36 hot spring sites (Figure 1). Each spring was sampled for both surface sediment and water. Sampling locations included Lhasa City (4 sites), Nyingchi City (5 sites), Shannan City (9 sites), and Shigatse City (18 sites). Based on different temperature gradients of the water, the hot springs were categorized into four types: GA (temperature ranging from 5°C to 40°C), GB (temperature ranging from 40°C to 50°C), GC (temperature ranging from 50°C to 60°C), and GD (temperature exceeding 60°C). Temperature is considered one of the key factors influencing the composition and ecological processes of hot spring communities (Oliverio et al., 2018). While some studies categorize temperature gradients into low and high temperature groups, we aim to provide a more detailed characterization of the impact of temperature on microbial communities by dividing the temperature gradient into four distinct categories (Wang et al., 2023). Therefore, we further refine the temperature gradient into four categories. During sediment sample collection, 25 mL of surface sediment (0–10 cm) was gathered in three separate batches from the outflow of each sampling site. These samples were placed in 50 mL sterile centrifuge tubes, covered with aluminum foil, and immediately frozen at −80°C for subsequent DNA extraction. For water sample collection, water was collected from the outflow of each sampling site using a 200 μm filter (to remove larger impurities) in three separate collections per site, yielding a total of 108 samples. The water samples were filtered using PC membranes (polycarbonate membrane, Millipore, United States) with a pore size of 0.22 μm to collect the DNA-containing material. These filtered PC membranes were placed in sterile cryogenic tubes, covered with aluminum foil, and immediately frozen at −80°C for preservation.

### 2.2 Determination of environmental physicochemical factors

The latitude (Lat), longitude (Lng), and altitude (ALT) of each sampling point were measured using the Global Positioning System (Explorist 500, Magellan, USA). On-site determination of environmental physicochemical factors for sediment primarily included pH, electrical conductivity (EC), sediment temperature (ST), and surface sediment moisture content (SM). The main methods involved the use of a portable soil pH/temperature meter (HANNA, HI99121, Italy) for measuring pH and ST of the hot spring surface sediment, a portable conductivity meter (HANNA, HI993310, Italy) for measuring EC, and a soil moisture analyzer (SIAS, SYS-SF, China) for determining SM of the surface sediment. Each physicochemical parameter was measured in triplicate. For on-site determination of water environmental physicochemical factors, measurements included pH, EC, water temperature (WT), turbidity (TUR), and ammonia nitrogen (NH_4_^+^-N). This involved the use of a high-precision portable multiparameter water quality analyzer (HANNA, HI98195, Italy) for measuring EC and pH, a high-precision turbidity analyzer (HANNA, HI98703, Italy) for TUR, a mercury thermometer for measuring WT, and a multiparameter water quality rapid analyzer (HANNA, HI83399, Italy) for measuring NH_4_^+^-N. Each physicochemical parameter was measured in triplicate. The actual measured values of each physicochemical factor are presented in Supplementary Table S1.

### 2.3 DNA extraction and amplicon sequencing

DNA was obtained from surface sediment samples of the hot springs using a DNA extraction kit (Novogene, China). DNA quality was evaluated using 1% agarose gel electrophoresis, and the concentration and purity of the extracted DNA were determined with a NanoDrop 2000 spectrophotometer (Thermo Fisher Scientific, Wilmington, DE, United States). Polymerase chain reaction (PCR) was conducted using primers 1391F (5-GTACACCGCCCGTC-3) and 1510R (5-TGATCCTTCTGCAGGTTCACCTAC-3) to amplify the V9 hypervariable region of the 18S rDNA. The PCR reaction mixture (50 μL) included 5 μL of 10× PCR buffer, 1.5 μL dNTPs, 1.5 μL each of the forward and reverse primer (10 μM), 0.5 μL Taq DNA polymerase (TaKaRa), 2 μL template DNA (5–30 ng), 1 μL Bovine Serum Albumin (BSA), and 37 μL ddH_2_O. The PCR protocol began with an initial denaturation at 94°C for 1 min, followed by 30 cycles of denaturation at 94°C for 20 s, annealing at 57°C for 25 s, extension at 68°C for 45 s, with a final extension step at 68°C for 10 min. The amplified products were sequenced and analyzed using the Ion S5 XL sequencing platform (Novogene, China). For extracting DNA from water samples gathered on PC membranes, the Power Soil DNA Isolation Kit (Qiagen, Germantown, MD, USA) was employed. Following DNA quality, concentration, and purity assessment, PCR amplification was performed using the same primers as used for sediment, and the resulting amplified products were sequenced and analyzed on the Ion S5 XL sequencing platform (Novogene, China).

### 2.4 Data analysis and statistical processing

The original sequences were subjected to sequence quality control, dereplication, and chimera removal using QIIME 2 to obtain high-quality sequences, ultimately resulting in Amplicon Sequence Variants (ASVs) (Bolyen et al., 2019). R software was employed to annotate these ASVs using the SILVA database (version 138) (Quast et al., 2013). ASVs with confidence values lower than 0.8 were excluded, retaining the annotated ASVs after standardization for downstream analysis (Liu et al., 2020). Calculate the *α*-diversity for each sample, including the ACE and Richness indices, using the R package “vegan.” For Faith’s phylogenetic diversity (PD), use the R package “picante.” Perform Variation Partitioning Analysis (VPA) and Canonical Correspondence Analysis (CCA) using the R package “vegan” to investigate the environmental factors influencing community differences across various habitats and temperature gradients. Using the “ape” package, Principal Coordinate Analysis (PCoA) and Permutational Multivariate Analysis of Variance (PERMANOVA) were conducted based on Bray-Curtis distances to assess differences in species composition across various habitats and temperature gradients. Calculate Nearest Taxon Index (pNST), *β*-nearest taxon index (βNTI), Bray-Curtis-based Raup-Crick measure (RC_bray_), Nearest Taxon Index (NTI), Net Relatedness Index (NRI), standardized effect size of mean nearest taxon distance (SES.MNTD), and standardized effect size of mean pairwise phylogenetic distance (SES.MPD) using the R package “NST” (Zhou and Ning, 2017). Conduct co-occurrence network analysis and calculate natural connectivity using the R packages “psych” and “igraph,” and visualize the co-occurrence network in Gephi (version 0.9.2). Perform key species analysis using the R package “microeco.” Evaluate network stability using positive co-occurrence, negative co-occurrence, and the ratio of negative to positive co-occurrence (Herren and McMahon, 2017). Visualize the correlation between environmental factors and positive co-occurrence, negative co-occurrence, and the ratio of negative to positive co-occurrence using the R package “pheatmap.” All analyses were conducted in R-4.3.1. Plot sample distribution maps using ArcGIS 10.6.1.

## 3 Results

### 3.1 Microeukaryotic community composition and diversity in sediments and water

**Figure 2:**
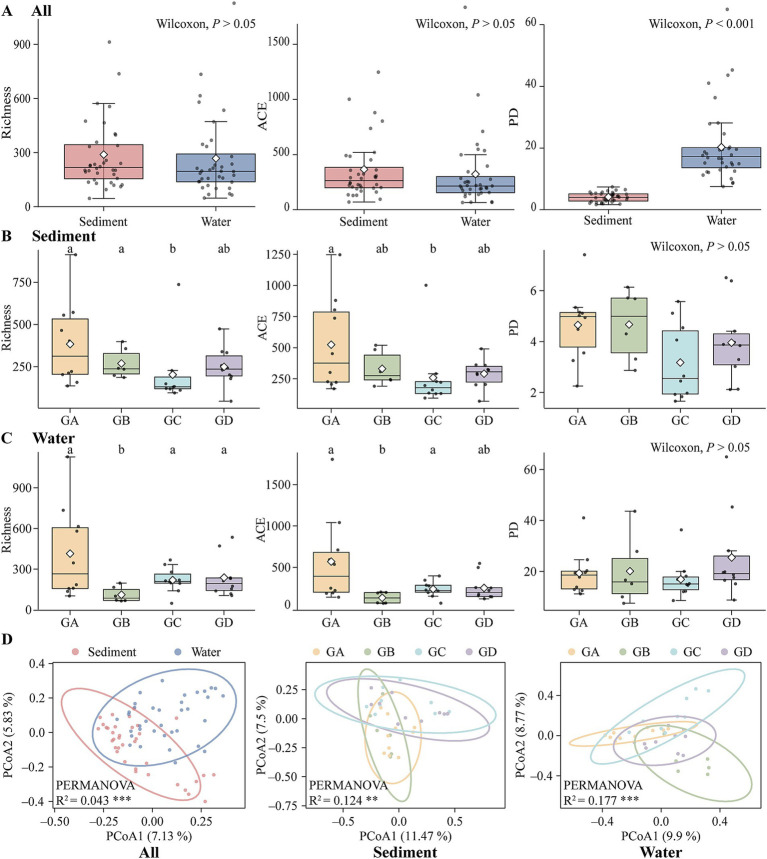
Sediment and water biodiversity and Principal Coordinates Analysis. **(A)** All. **(B)** Sediment. **(C)** Water. **(D)** PCoA analysis. (*p < 0.05; **p < 0.01; ***p < 0.001).

In this study, high-throughput sequencing of 18S rDNA was conducted on sediment and water samples collected from hot springs in southern Tibet. Following quality filtering, a sum of 9,118 high-quality ASVs were obtained. Among these, 6,675 ASVs could be classified at the phylum level (Supplementary Figure S1A). Overall, the top three dominant phyla in the sediment were Arthropoda (19.42% relative abundance), Ciliophora (9.91%), and Ascomycota (6.49%). Among these, the dominant phylum in the GA was Arthropoda (17.55%). In the GB, the top dominant phylum was Gastrotricha (21.70%). In the GC, the dominant phylum was Arthropoda (36.29%). In the GD, the top dominant phylum was Arthropoda (12.49%). In the water samples, the top three dominant phyla were Ochrophyta (16.90%), Arthropoda (11.15%), and Ascomycota (10.46%). Specifically, the top dominant phylum in the GA was Basidiomycota (22.83%). In the GB, the top dominant phylum was Ochrophyta (39.53%). In the GC, the top dominant phylum was Ochrophyta (18.17%). In the GD, the top dominant phylum was Arthropoda (Supplementary Figure S1B; 25.72%). The *α*-diversity (ACE, Richness) of sediments was significantly higher than that of the water (Figure 2A; Wilcoxon; *p* > 0.05). Under different temperature gradients, the richness index of sediment samples decreased with increasing temperature. The richness index of GA and GB was markedly greater than that of GC (Wilcoxon; *p* < 0.05). The ACE index followed a similar trend to the richness index with increasing temperature, with GA’s ACE index being significantly higher than that of GC (Figure 2B; Wilcoxon, *p* < 0.05). For the water samples, the richness of GB was significantly lower than that of the other three groups (Figure 2C; Wilcoxon, *p* < 0.05). The ACE index showed a similar trend to the richness index with increasing temperature, with GB’s ACE index being significantly lower than that of GA and GC (Wilcoxon; *p* < 0.05). PCoA and PERMANOVA analysis indicated significant differences in composition of microeukaryotic communities among different habitats (Figure 2D; *R*^2^ = 0.043; *p* < 0.001). Under different temperature gradients, there were significant differences in the microeukaryotic community composition of sediment samples (*R*^2^ = 0.124; *p* < 0.01) and water samples (*R*^2^ = 0.177; *p* < 0.001).

We were surprised to find that the phylogenetic diversity of water is significantly higher than that of the sediments (Figure 2A; Wilcoxon; *p* < 0.001). However, there were no significant differences within the sediment or water groups. Given that the phylogenetic diversity (PD) of sediments is significantly lower than that of water, we further explored the phylogenetic patterns using NTI, NRI, SES.MNTD and SES.MPD to understand their phylogenetic patterns. We used SES.MPD to measure the phylogenetic relatedness between species (i.e., clustering, overdispersion, or randomness) (Kim and Lee, 2021). SES.MPD and SES.MNTD are equivalent to the reciprocal of NRI and NTI. Water had a higher clustering (mean NTI = 2.134) compared to sediments (Supplementary Figure S2A; mean NTI = 0.875; ANOVA; *p* < 0.001). The SES.MNTD of sediments (mean = − 0.875) was significantly greater than that of water (Supplementary Figure S2A; mean = − 2.134; ANOVA, *p* < 0.001). There were no significant differences in NRI and SES.MPD between sediments and water. Under different temperature gradients, there were no significant differences in any of the four indices within the sediment group, with both NTI and NRI being >0, indicating clustering in all four groups (Supplementary Figure S2B). In water group, NTI increased with temperature from GA to GB and then leveled off, remaining >0 overall, with NTI in GA significantly higher than in GB (Supplementary Figure S2C; DUNCAN, *p* < 0.05). SES.MNTD increased with temperature from GA to GB and then decreased and leveled off, with GB’s NTI significantly higher than GA’s (DUNCAN, *p* < 0.05). There were no significant differences in NRI and SES.MPD between groups, but a portion of NRI in GC was <0; however, the overall pattern still indicated clustering.

### 3.2 The co-occurrence patterns within the microeukaryotic communities in hot springs

**Figure 3:**
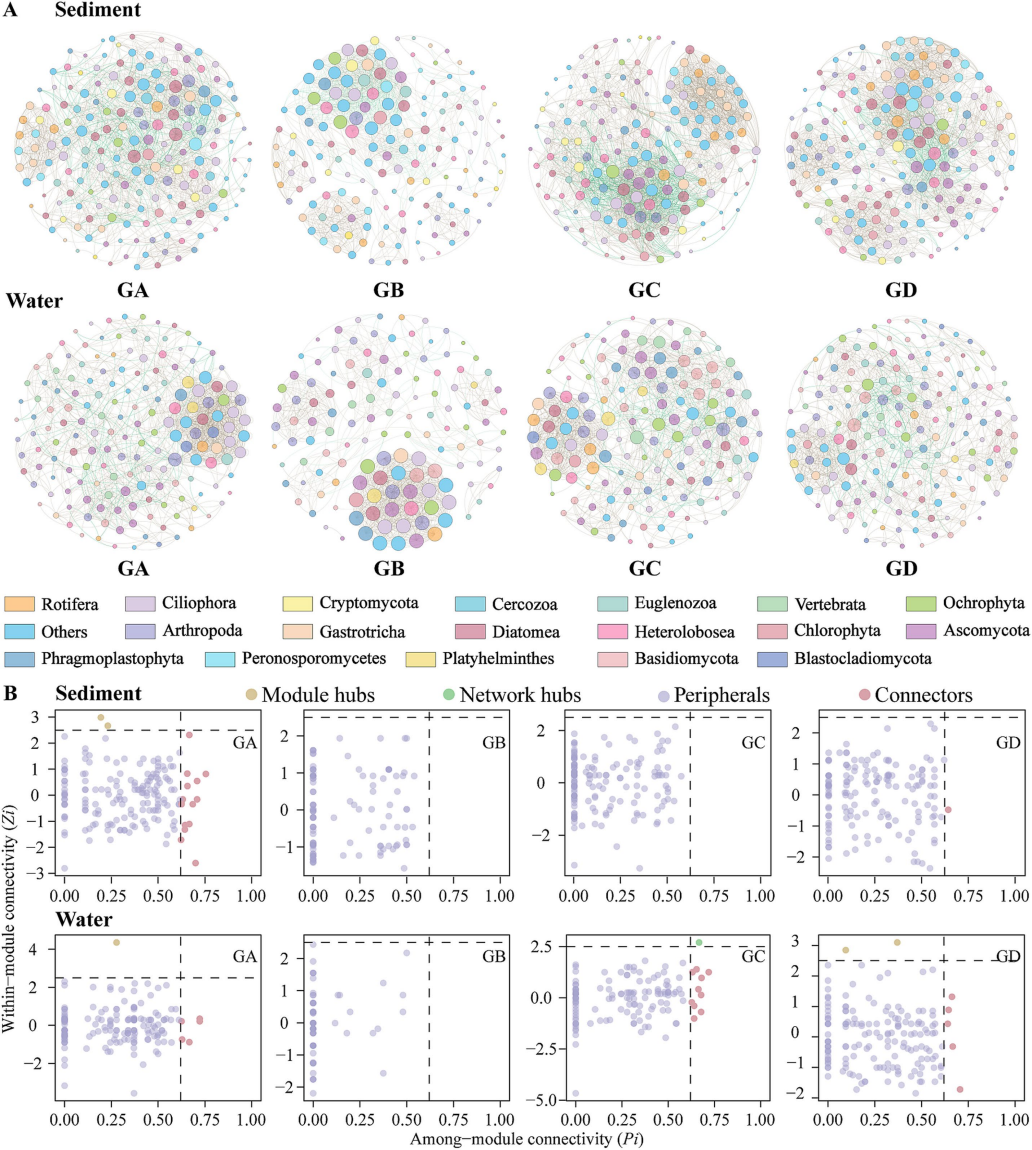
Co-occurrence network analysis. **(A)** Co-occurrence networks of all groups. **(B)** Keystone species analysis. Node size represents node degree; brown and green edges indicate positive and negative correlations between paired OTUs, respectively.

Co-occurrence networks were created using the top 200 ASVs selected based on their relative abundances in sediment and water (Figure 3A). The sediment co-occurrence network comprised 200 nodes connected by 858 edges, while the water network consisted of 200 nodes connected by 378 edges (Supplementary Table S2). Positive interactions among species dominated in the sediment network (94.29%), all species interactions in the water network were positive (100.00%). All network *R*^2^ values are >0 and comply with the power-law model. The small-world coefficients are all >1, and the topological parameters of the empirical networks exceed those of the random networks, showing that all networks exhibit small-world properties. The modularity of the empirical networks is greater than Modularity_r_ and > 0.4 (Liu et al., 2020; Ye et al., 2020), indicating that all networks are modular (Supplementary Table S2). Regardless of habitat or temperature gradients, microeukaryotic co-occurrence networks are not random but instead possess a hierarchical structure, being scale-free, modular, and small-world. Compared to sediments, the water networks have a smaller average path length and network diameter, but a larger average clustering coefficient and small-world coefficient (Supplementary Table S2). This suggests that microeukaryotic communities in water are more closely connected, while the sediment networks have higher density and average degree, indicating stronger connectivity between ASVs in sediment networks. In the sediment group, GC has higher density and average degree than other groups, and the connectivity between ASVs in the GC network is stronger. The average clustering coefficient and small-world coefficient of GB are higher than those of other groups, showing closer relationships within the GB network (Supplementary Table S2). In the water group, GC also has higher density and average degree than other groups, indicating stronger ASV connectivity in the GC network (Supplementary Table S2).

Based on the modular within-connectivity (Zi) and modular between-connectivity (Pi) of individual nodes, nodes are classified into four categories: Peripheral (Zi < 2.5, Pi <0.62), Connector (Zi < 2.5, Pi >0.62), Module Hub (Zi > 2.5, Pi <0.62), and Network Hub (Zi > 2.5, Pi >0.62) (Olesen et al., 2007). In the field of ecology, it is believed that peripherals may represent specialists, while module hubs and connectors resemble generalists, with network hubs acting as supergeneralists (Olesen et al., 2007). In the sediment, there were 2 connectors (Supplementary Figure S3B). The GA had 15 connectors and 2 module hubs (Figure 3B). The GD had 1 connector. In the water, such keystone species were lacking (Supplementary Figure S3B), but the GA had 5 connectors and 1 module hub. The GC had 10 connectors and 1 network hub. The GD had 5 connectors and 2 module hubs (Figure 3B).

Although there was no significant difference in positive cohesion and negative cohesion between sediment and water, the ratio of negative cohesion to positive cohesion (absolute N/P cohesion) differed significantly (Supplementary Figure S4A; Wilcoxon; *p* < 0.001). In the sediment group, GA significantly differed from GB and GD in absolute N/P cohesion (Supplementary Figure S4C; Wilcoxon; *p* < 0.05), with both GB and GD predominantly exhibiting negative associations. As the temperature increases, absolute N/P cohesion gradually increases. In the water group, GA and GB significantly differed from GD in absolute N/P cohesion (Supplementary Figure S4C; Wilcoxon, *p* < 0.05), with GA and GB predominantly exhibiting negative associations. As the temperature increases, absolute N/P cohesion gradually decreases.

### 3.3 Community assembly and driving factors of eukaryotic microeukaryotic in hot springs

**Figure 4:**
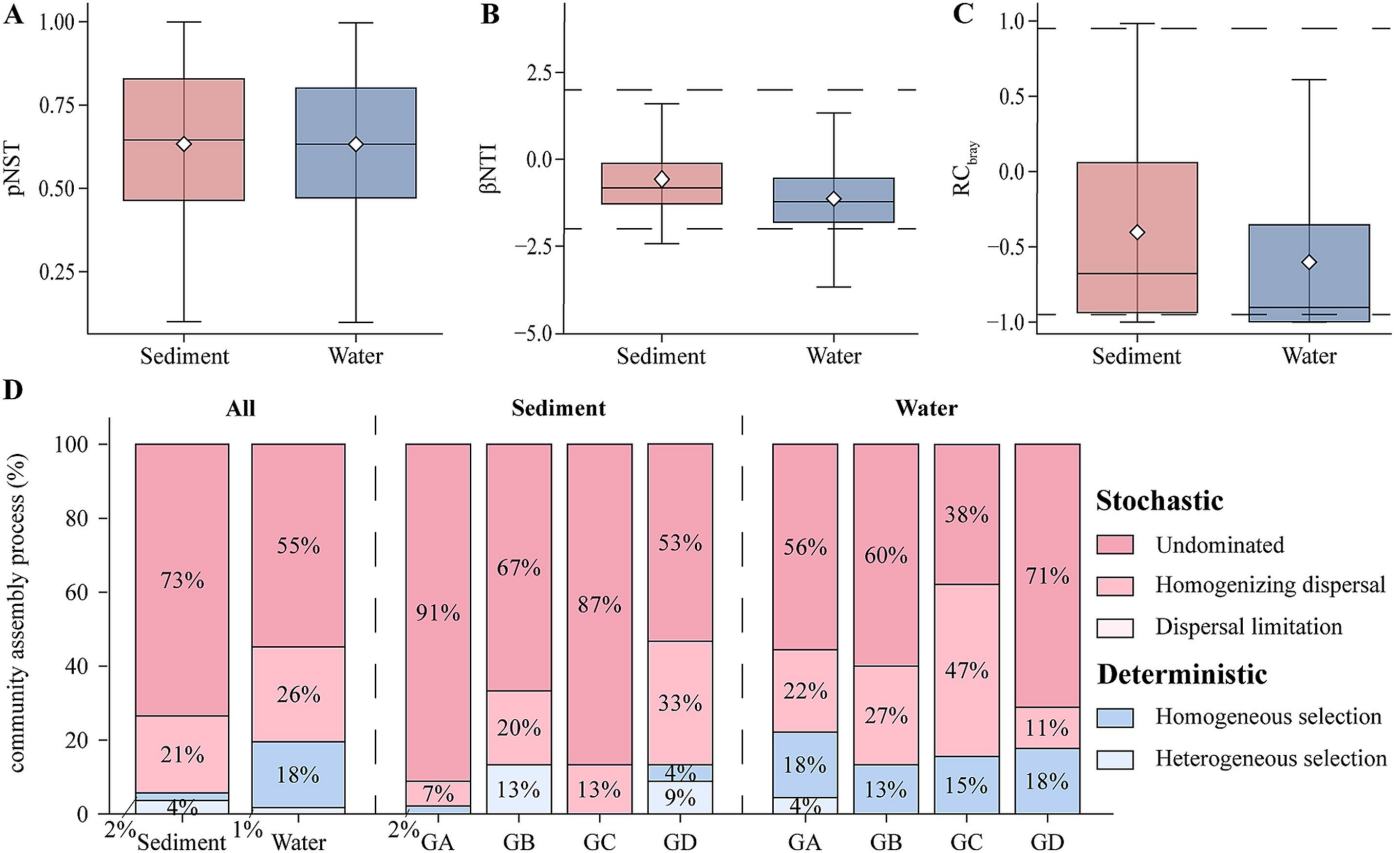
The construction process of microeukaryotic communities. **(A)** Comparison of pNST in sediment and water. **(B)** Comparison of βNTI in sediment and water. **(C)** Comparison of RC_bray_ in sediment and water. **(D)** Null model illustrating the contributions of different ecological processes to the composition of microeukaryotic communities.

This study analyzed the primary mechanisms governing community assembly processes using null model. Based on the analysis from the null model, computations of the phylogenetic pNST, βNTI and RC_bray_ were conducted (Figures 4A–C). The results indicate a predominant role of stochastic processes in both sediment and water samples (|βNTI| ≤ 2), accounting for 94% in sediment and 81% in water samples (Figure 4D). Further analysis using RC_bray_ revealed that in sediments, deterministic processes account for only 6%, while the remaining 94% are driven by stochastic processes. Among these, undominated processes (|RC_bray_| ≤ 0.95) dominate at 73%, followed by homogeneous selection (RC_bray_ < − 0.95) at 21% (Figure 4D). In the sediment group, the proportion of undominated processes gradually decreases with rising temperatures. Similarly, in the water group, deterministic processes account for only 19%, with stochastic processes mainly influenced by undominated processes (55%) and homogeneous selection (Figure 4D; 26%). Overall, undominated processes are the primary factors influencing the assembly of microeukaryotic communities in hot springs.

**Figure 5:**
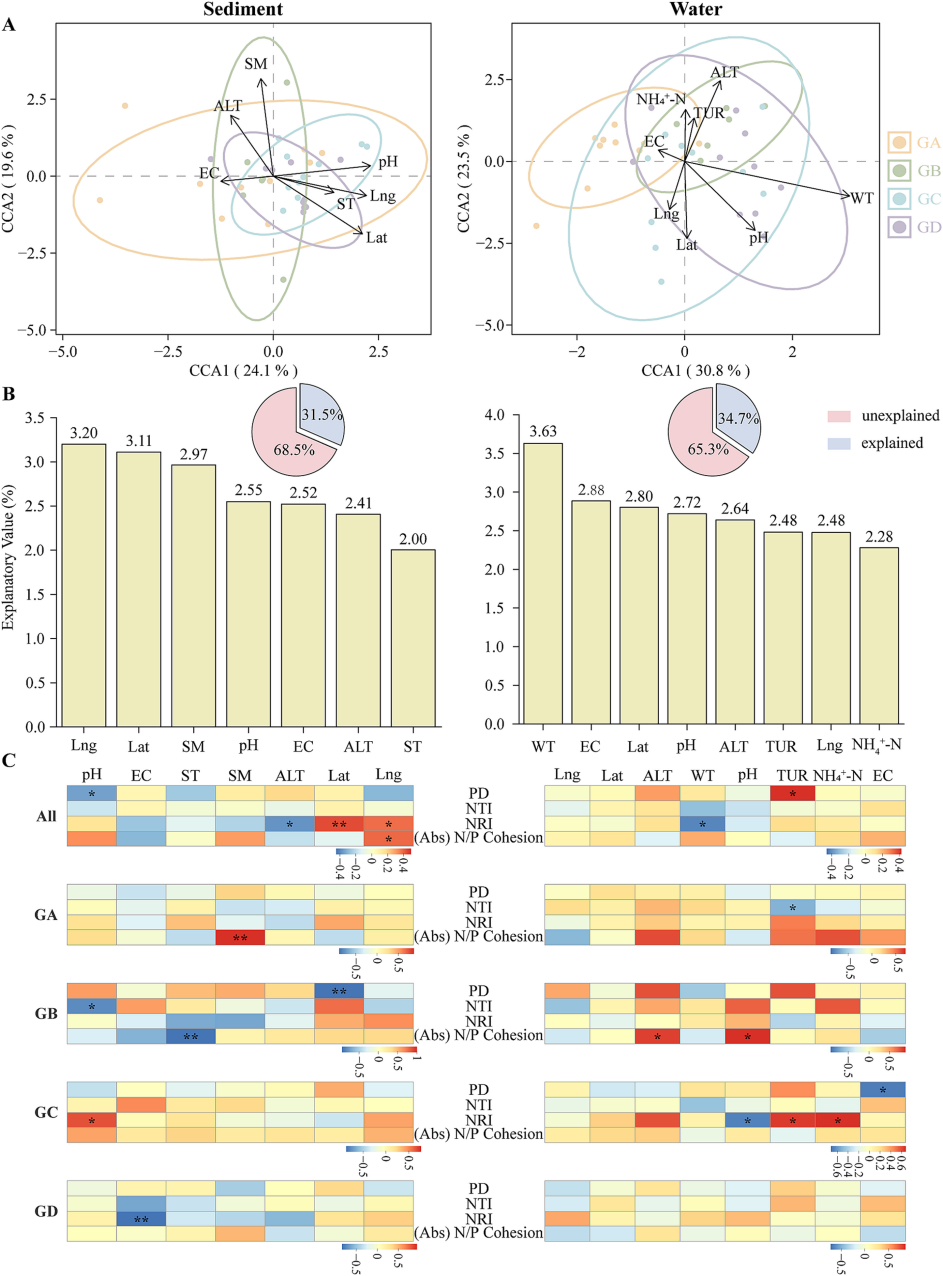
The environmental factors influencing the eukaryotic microbiome. **(A)** Canonical Correspondence Analysis (CCA) of eukaryotic communities and environmental factors. **(B)** Variation partitioning analysis (VPA) of microeukaryotic communities by environmental factors. **(C)** Spearman correlation analysis between microeukaryotic communities and environmental factors in hot springs. EC, electrical conductivity; ST, sediment temperature; SM, surface sediment moisture content; ALT, altitude; Lat, latitude; Lng, longitude; WT, water temperature; TUR, turbidity; NH_4_^+^-N, ammonia nitrogen. (*p < 0.05; **p < 0.01; ***p < 0.001).

Since the maximum gradient on the ordination axis exceeded 3, CCA analysis was selected. In sediment, SM and pH were identified as the primary environmental factors influencing the distribution of microeukaryotes, with Lng and Lat also showing significant effects (Figure 5A). In water, WT was the key environmental factor affecting microeukaryote distribution, while ALT, pH, and Lat also played important roles (Figure 5A). VPA was employed to assess the contribution of environmental factors to microbial community variation. In sediment, environmental factors explained 31.5% of community variation, with longitude, latitude, and SM as the major influencers (Figure 5B). In water, environmental factors accounted for 34.7% of community variation, with WT, EC, and Lat as the principal factors (Figure 5B). To explore the relationship between microeukaryotic community structure and environmental factors, we conducted correlation analyses between microeukaryotic community parameters (including phylogenetic patterns and absolute N/P cohesion) and environmental factors. In this study, community parameters in the water column were generally negatively correlated with environmental factors. Specifically, WT demonstrated a negative correlation with NRI (Figure 5C; *p* < 0.05), whereas PD demonstrated a positive correlation with TUR (*p* < 0.05). For sediments, pH showed a negative correlation with PD (Figure 5C; *p* < 0.05), Lat exhibited a positive correlation with NRI (*p* < 0.01), and Lng was positively correlated with absolute N/P cohesion (*p* < 0.05). In the GA of sediments, SM had a positive correlation with absolute N/P cohesion (*p* < 0.01). In the GB, absolute N/P cohesion had a negative relationship with ST (p < 0.01), and PD had a negative relationship with Lat (*p* < 0.01). In the GC, pH was positively correlated with NRI (*p* < 0.05). In the GD, EC was negatively correlated with NRI (*p* < 0.01). For the water, in the GA, TUR was negatively correlated with NTI (Figure 5C; *p* < 0.05). In the GB, absolute N/P cohesion was positively correlated with ALT and pH (*p* < 0.05). In the GC, NRI was negatively correlated with pH (*p* < 0.05), and NRI was positively correlated with TUR and NH_4_^+^-N (*p* < 0.05). EC was negatively correlated with PD (*p* < 0.05), while no significant correlations were found in the GD.

## 4 Discussion

### 4.1 The community structure of sediment and water communities

Both habitat changes and rising temperatures lead to shifts in the composition and diversity of eukaryotic microeukaryotic communities. In this study, the dominant phylum of microeukaryotes in geothermal spring waters in the southern Tibetan Plateau are Arthropoda, Ascomycota, Ochrophyta, Ciliophora, and Rotifera. Similarly, research conducted on hot springs in the Taupō Volcanic Zone of New Zealand has demonstrated a dominance of Ciliophora and Ochrophyta (Oliverio et al., 2018). In both the GD of sediments and water, Arthropoda is the dominant phylum. Arthropods possess a special protein called HSP (heat shock protein), which repairs heat stress-induced cellular damage by adding an extra protective layer (Benoit et al., 2019). Besides HSP, other cellular components in Ochrophyta may also contribute to temperature adaptation (Collén et al., 2007). It is generally believed that species composition results from environmental (abiotic) and competitive exclusion (biotic) filters (Weiher et al., 1998; Belyea and Lancaster, 1999; Silvertown, 2004). Habitat differences between sediments and water lead to different species compositions, yet the *α*-diversity in sediments is not notably lower than in water, which is contrary to findings in other aquatic environments (Ren et al., 2022). This is due to the dynamic equilibrium between water and sediments (Hou et al., 2013), leading to similar diversities in both environments. The highest α-diversity is observed in the GA for both sediments and water, likely because the broader temperature range in GA allows for more species accommodation. Faith’s PD is considered the minimum total length of all existing phylogenetic branches required to cover all taxa in a given phylogenetic tree (Faith, 1992; Faith and Baker, 2006). The PD in sediments is significantly lower than in water, which differs from observations in Tengchong hot springs (He et al., 2021). This indicates that water communities possess higher phylogenetic diversity compared to sediment communities, reflecting more complex evolutionary relationships and a more dispersed branching pattern, and suggests that sediments have greater evolutionary physiological constraints, showing lower phylogenetic diversity (Qian et al., 2013).

Additionally, as shown in Supplementary Figure S2, the NTI values for water microeukaryotic communities are significantly higher than those for sediment microeukaryotic communities, indicating that the phylogenetic structure of water is more clustered (NTI > 0 and NRI > 0). In community networks, the relationships between taxa are influenced by two non-mutually exclusive mechanisms: species interactions and environmental filtering (Barberán et al., 2012; Freilich et al., 2018). Generally, environmental filtering leads to phylogenetic clustering, with most instances occurring among closely related species (Ginocchio et al., 2017). Phylogenetic overdispersion may be due to competitive behaviors (Webb et al., 2002; Manish and Pandit, 2018). The highest NTI in the water GA, with an average temperature range of 5–40°C, provides a broader and more stable environment with fewer disturbances, allowing more species to coexist and evolve, resulting in higher NTI values. SES.MNTD of sediment is significantly greater than that in water, with GB in the water group showing significantly higher SES.MNTD than other groups. MPD assesses the phylogenetic structure at deeper nodes, serving as an indicator of the pairwise phylogenetic distances between coexisting species and also reflecting divergence at the community or genus level (Cadotte and Davies, 2016; Webb, 2000). MNTD calculates the phylogenetic structure at shallower nodes, quantifying the phylogenetic distances at the terminal nodes between closest neighbors (sister taxa), describing the divergence at the species level (Cadotte and Davies, 2016). The shallow node species in GB of sediments and water are more ancient or unique in evolutionary terms, with greater phylogenetic distances from other species on the phylogenetic tree.

### 4.2 Patterns of community co-occurrence in sediments and water

In this study, the composition of microeukaryotic communities varied between sediment and water habitats, forming based on distinct rules (Ren et al., 2021) and creating unique networks. Although we cannot fully explain the biological interactions within microeukaryotic networks, these networks help us understand the complexity of communities and their responses to environmental changes (Qiu et al., 2021). By comparing the topological structures, we found that the sediment network had stronger and tighter interconnectivity. Sediments, being in relatively stable environments, contrast with water environments, which are more susceptible to disturbances from weather events and animal activities, making them dynamic systems. This difference may be attributed to the dynamic and unstable nature of the water environment, which is heavily influenced by subsurface hydrology and geological events s (Deng et al., 2010; Cox et al., 2015). In microeukaryotic networks, module hubs and connectors are crucial for maintaining ecosystem stability and facilitating the assembly mechanisms of microeukaryotic communities (Zhou et al., 2010; Banerjee et al., 2018). Their disappearance can lead to the disintegration of the entire network (Wang et al., 2021). In different habitats, GB lacked keystone species, and the water temperature in GB ranged from 40 to 50°C, whereas most microeukaryotes typically thrive at temperatures between 20 and 40°C, with thermophilic organisms preferring environments above 50°C (Turner et al., 2007). Cohesion is a measure of network stability, providing insight into the connectivity of microeukaryotic communities, and is hypothesized to be related to community dynamics (MacArthur, 1955; Nilsson and McCann, 2016). Increasing research indicates that networks with a higher absolute N/P cohesion respond more stably to environmental changes (Coyte et al., 2015; Neutel et al., 2002). Negative feedback can suppress disturbances to the community, while positive feedback amplifies disturbances (Fontaine et al., 2011). Therefore, sediment exhibits the highest stability, with the GB and GD sediment groups and the GA and GB water groups demonstrating higher stability. Both absolute N/P cohesion indicate that with rising temperatures, the stability of the sediment group increases, while the stability of the water group decreases. Sediments provide a relatively stable habitat, as microeukaryotic communities in these environments are influenced by long-term processes of accumulation, deposition, and erosion (Du et al., 2020). Low-temperature habitats are more favorable for colonization, while high-temperature habitats exhibit a pronounced “directional ecological filtering” effect (Alexander et al., 2011), where only species tolerant of high temperatures can survive, leading to greater resistance to disturbance and higher network robustness. In contrast, water is more susceptible to disturbances from weather events and animal activities, as microeukaryotes in the water are also influenced by rainwater, groundwater, and soil bacteria (Nelson, 2009; Sloan et al., 2006). The increased environmental pressure reduces the stability of microeukaryotic communities, possibly because thermophilic organisms gain an advantage over general microeukaryotes, leading to a decline in species diversity and, consequently, a reduction in network stability.

### 4.3 Microeukaryotic community assembly in geothermal hot springs is dominated by stochasticity

Gaining insight into the relative contributions of deterministic and stochastic processes in community assembly aids in revealing the ecological strategies of coexisting species (Kraft et al., 2015). The assembly of communities in both sediment and water is predominantly governed by undominant processes, which contrasts with He et al. (2021) study on Tengchong, Yunnan Province. In that research, water communities are primarily driven by stochasticity, while sediment communities are mainly influenced by deterministic processes. Several studies have demonstrated that low-abundance communities are primarily governed by undominant processes induced by weak selection/dispersal, diversification, and drift (Huang et al., 2022). Undominant processes imply that drift plays a larger role in community assembly (Stegen et al., 2015). Fungi, which make up a significant portion of the community, adapt to environmental stress through spore reproduction, producing a large number of spores (Ingold, 1971), and these spores are dispersed through physical media like water (Walters et al., 2022). During the process of community assembly, the signal of dispersal limitation is relatively weak, while the signal of homogenizing dispersal is strong, which contradicts the findings of Ren et al. (2022). In Ren’s study, the isolated nature of the hot karst lakes resulted in weak connectivity between lakes, significantly limiting the dispersal of microeukaryotes. However, in this study, the hot springs in southern Tibet are located in the Himalayan geothermal belt, where the sampling points are more connected. Additionally, the chemical properties of the hot spring waters are quite similar, leading to more regional similarities in community structure and stronger signals of homogenizing dispersal. Under the influence of strong environmental selection, communities often exhibit lower dispersal limitation. This is because environmental selection causes microeukaryotic communities to be primarily composed of a few highly abundant species, while the birth and death rates of rare species differ significantly (Putman et al., 2021).

Results from CCA and VPA indicate that the key influencing factors of hot spring sediment communities are Lat, Lng and SM. Although WT and Lat are also major factors influencing hot spring water communities, the dynamic environment and rapid water flow may overwhelm deterministic processes. Discrete boundaries and upward flow paths impose significant constraints on the dispersal of microeukaryotic communities; however, increased drift can promote the introduction of stochasticity into these communities (Stegen et al., 2013; Dini-Andreote et al., 2015). Temperature is a key factor regulating microeukaryotic metabolism and growth, as it promotes the dissolution of mineral elements, thereby accelerating reaction rates (Konrad-Schmolke et al., 2018). The Metabolic Niche Hypothesis suggests that only taxa with lifestyles capable of maintaining sufficient metabolic energy can survive in extreme environments (Sharp et al., 2014; Clarke and Gaston, 2006). VPA was employed to evaluate the relative effects of environmental selection and spatial factors on the structure of microeukaryotic communities (Smith and Lundholm, 2010). The low explanatory power of each factor in both sediment and water may be attributed to the complex and variable nature of the hot spring ecosystem. Other studies indicate that VPA cannot quantify the impact of microeukaryotic symbiosis on community distribution (Lima-Mendez et al., 2015; Wei et al., 2016). Temperature increases promote speciation and random mortality (He et al., 2022), thereby reducing network stability. EC reflects the concentration of dissolved salts in sediments, playing an important role in microeukaryotic respiration, carbon metabolism, and growth (Rath and Rousk, 2015), directly influencing microeukaryotic metabolism and other life activities (Decamp et al., 2003; Thompson et al., 2017).

## 5 Conclusion

This study investigated the community structure, phylogenetic patterns, co-occurrence relationships, and community assembly mechanisms of microeukaryotes in geothermal springs across different habitats and temperature gradients in southern Tibet. We found that *α* diversity was highest at lower temperatures, with stronger phylogenetic clustering in water communities. In both sediment and water groups, the shallow-node species in GB were evolutionarily more ancient or unique, with greater phylogenetic distances from other species in the phylogenetic tree. The sediment network was more complex, exhibiting stronger and tighter interconnectivity. As temperature increased, the network stability of sediment communities improved, whereas the stability of water networks decreased. Both sediment and water communities were mainly influenced by stochastic processes, with longitude and latitude being the main driver of sediment community variation, while T and EC were the key factors influencing water communities. In summary, our findings enhance the understanding of the maintenance mechanisms of microeukaryotes under varying habitats and temperature gradients and their resilience to external disturbances in extreme environments. This research provides a theoretical basis for future studies on the diversity and conservation of geothermal microeukaryotes.

## Data Availability

The original contributions presented in the study are included in the supplementary material, further inquiries can be directed to the corresponding authors.

## References

[ref1] Alexander J. M. Kueffer C. Daehler C. C. Edwards P. J. Pauchard A. Seipel T.. (2011). Assembly of nonnative floras along elevational gradients explained by directional ecological filtering. Proc. Natl. Acad. Sci. U. S. A. 108, 656–661. doi: 10.1073/pnas.1013136108, PMID: 21187380 PMC3021079

[ref2] Bahram M. Kohout P. Anslan S. Harend H. Abarenkov K. Tedersoo L. (2016). Stochastic distribution of small soil eukaryotes resulting from high dispersal and drift in a local environment. ISME J. 10, 885–896. doi: 10.1038/ismej.2015.164, PMID: 26394006 PMC4796928

[ref3] Banerjee S. Schlaeppi K. van der Heijden M. G. A. (2018). Keystone taxa as drivers of microbiome structure and functioning. Nat. Rev. Microbiol. 16, 567–576. doi: 10.1038/s41579-018-0024-1, PMID: 29789680

[ref4] Barberán A. Bates S. T. Casamayor E. O. Fierer N. (2012). Using network analysis to explore co-occurrence patterns in soil microbial communities. ISME J. 6, 343–351. doi: 10.1038/ismej.2011.119, PMID: 21900968 PMC3260507

[ref5] Belyea L. R. Lancaster J. (1999). Assembly rules within a contingent ecology. Oikos 86, 402–416. doi: 10.2307/3546646

[ref6] Benoit J. B. Lazzari C. R. Denlinger D. L. Lahondère C. (2019). Thermoprotective adaptations are critical for arthropods feeding on warm-blooded hosts. Curr. Opin. Insect Sci. 34, 7–11. doi: 10.1016/j.cois.2019.02.003, PMID: 31247421

[ref7] Berdjeb L. Parada A. Needham D. M. Fuhrman J. A. (2018). Short-term dynamics and interactions of marine protist communities during the spring-summer transition. ISME J. 12, 1907–1917. doi: 10.1038/s41396-018-0097-x, PMID: 29599520 PMC6052004

[ref8] Bolyen E. Rideout J. R. Dillon M. R. Bokulich N. A. Abnet C. C. Al-Ghalith G. A.. (2019). Reproducible, interactive, scalable and extensible microbiome data science using QIIME 2. Nat. Biotechnol. 37, 852–857. doi: 10.1038/s41587-019-0209-9, PMID: 31341288 PMC7015180

[ref9] Cadotte M. W. Davies T. J. (2016). Phylogenies in ecology: A guide to concepts and methods. New Jersey: Princeton University Press.

[ref10] Chase J. M. Myers J. A. (2011). Disentangling the importance of ecological niches from stochastic processes across scales. Philos. Trans. R. Soc. B: Biol. Sci. 366, 2351–2363. doi: 10.1098/rstb.2011.0063, PMID: 21768151 PMC3130433

[ref11] Clarke A. Gaston K. J. (2006). Climate, energy and diversity. Proc. R. Soc. B: Biol. Sci. 273, 2257–2266. doi: 10.1098/rspb.2006.3545, PMID: 16928626 PMC1636092

[ref12] Clauset A. Moore C. Newman M. E. J. (2008). Hierarchical structure and the prediction of missing links in networks. Nature 453, 98–101. doi: 10.1038/nature06830, PMID: 18451861

[ref13] Collén J. Guisle-Marsollier I. Léger J. J. Boyen C. (2007). Response of the transcriptome of the intertidal red seaweed *Chondrus crispus* to controlled and natural stresses. New Phytol. 176, 45–55. doi: 10.1111/j.1469-8137.2007.02152.x, PMID: 17803640

[ref14] Cox S. C. Menzies C. D. Sutherland R. Denys P. H. Chamberlain C. Teagle D. A. H. (2015). Changes in hot spring temperature and hydrogeology of the alpine fault hanging wall, New Zealand, induced by distal South Island earthquakes. Geofluids 15, 216–239. doi: 10.1111/gfl.12093

[ref15] Coyte K. Z. Schluter J. Foster K. R. (2015). The ecology of the microbiome: networks, competition, and stability. Science 350, 663–666. doi: 10.1126/science.aad2602 26542567

[ref16] Decamp O. Cody J. Conquest L. Delanoy G. Tacon A. G. J. (2003). Effect of salinity on natural community and production of *Litopenaeus vannamei* (Boone), within experimental zero-water exchange culture systems. Aquac. Res. 34, 345–355. doi: 10.1046/j.1365-2109.2003.00842.x

[ref17] Deng J. Xiao C. H. Wang Q. F. Zhou X. Z. Yang L. Q. Zhang J.. (2010). Influence of the Chuxiong Yao’an earthquake on the mineralization of Hot Springs in the Tengchong geothermal area, southwestern China. Acta Geol. Sinica Engl. Ed. 84, 1391–1400. doi: 10.1111/j.1755-6724.2010.00349.x, PMID: 39810616

[ref18] Dini-Andreote F. Stegen J. C. van Elsas J. D. Salles J. F. (2015). Disentangling mechanisms that mediate the balance between stochastic and deterministic processes in microbial succession. Proc. Natl. Acad. Sci. U. S. A. 112, E1326–E1332. doi: 10.1073/pnas.1414261112, PMID: 25733885 PMC4371938

[ref19] Du L. Wang R. Gao X. Hu Y. X. Guo S. L. (2020). Divergent responses of soil bacterial communities in erosion-deposition plots on the loess plateau. Geoderma 358:113995. doi: 10.1016/j.geoderma.2019.113995

[ref20] Faith D. P. (1992). Conservation evaluation and phylogenetic diversity. Biol. Conserv. 61, 1–10. doi: 10.1016/0006-3207(92)91201-3

[ref21] Faith D. P. Baker A. M. (2006). Phylogenetic diversity (PD) and biodiversity conservation: some bioinformatics challenges. Evol. Bioinforma. 2, 121–128. doi: 10.1177/117693430600200007, PMID: 19455206 PMC2674678

[ref22] Fargione J. Brown C. S. Tilman D. (2003). Community assembly and invasion: an experimental test of neutral versus niche processes. Proc. Natl. Acad. Sci. U. S. A. 100, 8916–8920. doi: 10.1073/pnas.1033107100, PMID: 12843401 PMC166413

[ref23] Farrell H. L. Léger A. Breed M. F. Gornish E. S. (2020). Restoration, soil organisms, and soil processes: emerging approaches. Restor. Ecol. 28, 307–310. doi: 10.1111/rec.13237, PMID: 39810616

[ref24] Faust K. Raes J. (2012). Microbial interactions: from networks to models. Nat. Rev. Microbiol. 10, 538–550. doi: 10.1038/nrmicro2832, PMID: 22796884

[ref25] Fontaine C. Guimarães P. R. Kéfi S. Loeuille N. Memmott J. van der Putten W. H.. (2011). The ecological and evolutionary implications of merging different types of networks. Ecol. Lett. 14, 1170–1181. doi: 10.1111/j.1461-0248.2011.01688.x, PMID: 21951949

[ref26] Freilich M. A. Wieters E. Broitman B. R. Marquet P. A. Navarrete S. A. (2018). Species co-occurrence networks: can they reveal trophic and non-trophic interactions in ecological communities? Ecology 99, 690–699. doi: 10.1002/ecy.2142, PMID: 29336480

[ref27] Ginocchio R. León-Lobos P. Arellano E. C. Anic V. Ovalle J. F. Baker A. J. M. (2017). Soil physicochemical factors as environmental filters for spontaneous plant colonization of abandoned tailing dumps. Environ. Sci. Pollut. Res. 24, 13484–13496. doi: 10.1007/s11356-017-8894-8, PMID: 28390018

[ref28] Guseva K. Darcy S. Simon E. Alteio L. V. Montesinos-Navarro A. Kaiser C. (2022). From diversity to complexity: microbial networks in soils. Soil Biol. Biochem. 169:108604. doi: 10.1016/j.soilbio.2022.108604, PMID: 35712047 PMC9125165

[ref29] He J. K. Lin H. X. Wang R. X. Dai C. Yu H. Y. Tu J. H.. (2022). Joint effects of environmental filtering and dispersal limitation on the species assemblage of the Tibetan plateau. J. Biogeogr. 49, 640–653. doi: 10.1111/jbi.14328

[ref30] He Q. Wang S. Hou W. G. Feng K. Li F. R. Hai W. M.. (2021). Temperature and microbial interactions drive the deterministic assembly processes in sediments of hot springs. Sci. Total Environ. 772:145465. doi: 10.1016/j.scitotenv.2021.145465, PMID: 33571767

[ref31] Hernandez D. J. David A. S. Menges E. S. Searcy C. A. Afkhami M. E. (2021). Environmental stress destabilizes microbial networks. ISME J. 15, 1722–1734. doi: 10.1038/s41396-020-00882-x, PMID: 33452480 PMC8163744

[ref32] Herren C. M. McMahon K. D. (2017). Cohesion: a method for quantifying the connectivity of microbial communities. ISME J. 11, 2426–2438. doi: 10.1038/ismej.2017.91, PMID: 28731477 PMC5649174

[ref33] Hochstein M. P. Regenauer-Lieb K. (1998). Heat generation associated with collision of two plates: the Himalayan geothermal belt. J. Volcanol. Geotherm. Res. 83, 75–92. doi: 10.1016/S0377-0273(98)00018-3

[ref34] Hou W. G. Wang S. Dong H. L. Jiang H. C. Briggs B. R. Peacock J. P.. (2013). A comprehensive census of microbial diversity in Hot Springs of Tengchong, Yunnan Province China using 16S rRNA gene pyrosequencing. PLoS One 8:e53350. doi: 10.1371/journal.pone.0053350, PMID: 23326417 PMC3541193

[ref35] Hu Y. A. Cheng H. F. Tao S. (2022). Opportunity and challenges in large-scale geothermal energy exploitation in China. Crit. Rev. Environ. Sci. Technol. 52, 3813–3834. doi: 10.1080/10643389.2021.1971004

[ref36] Huang L. B. Bai J. H. Wang J. J. Zhang G. L. Wang W. Wang X.. (2022). Different stochastic processes regulate bacterial and fungal community assembly in estuarine wetland soils. Soil Biol. Biochem. 167:108586. doi: 10.1016/j.soilbio.2022.108586

[ref37] Ingold C. T. (1971). Fungal spores: Their liberation and dispersal. London: Clarendon Press.

[ref38] Jeroen R. Peer B. (2008). Molecular eco-systems biology: towards an understanding of community function. Nat. Rev. Microbiol. 6, 693–699. doi: 10.1038/nrmicro1935, PMID: 18587409

[ref39] Kim H. Lee C. B. (2021). On the relative importance of landscape variables to plant diversity and phylogenetic community structure on uninhabited islands, South Korea. Landscape Ecol. 36, 209–221. doi: 10.1007/s10980-020-01134-1

[ref40] Konrad-Schmolke M. Halama R. Wirth R. Thomen A. Klitscher N. Morales L.. (2018). Mineral dissolution and reprecipitation mediated by an amorphous phase. Nat. Commun. 9:1637. doi: 10.1038/s41467-018-03944-z, PMID: 29691391 PMC5915427

[ref41] Kraft N. J. B. Adler P. B. Godoy O. James E. C. Fuller S. Levine J. M. (2015). Community assembly, coexistence and the environmental filtering metaphor. Funct. Ecol. 29, 592–599. doi: 10.1111/1365-2435.12345

[ref42] Kublanov I. V. Perevalova A. A. Slobodkina G. B. Lebedinsky A. V. Bidzhieva S. K. Kolganova T. V.. (2008). Biodiversity of thermophilic prokaryotes with hydrolytic activities in Hot Springs of Uzon caldera, Kamchatka (Russia). Appl. Environ. Microb. 75, 286–291. doi: 10.1128/AEM.00607-08, PMID: 18978089 PMC2612224

[ref43] Langner H. W. Jackson C. R. McDermott T. R. Inskeep W. P. (2001). Rapid oxidation of Arsenite in a hot spring ecosystem, Yellowstone National Park. Environ. Sci. Technol. 35, 3302–3309. doi: 10.1021/es0105562, PMID: 11529568

[ref44] Li L. W. Ma Z. S. (2020). Species sorting and neutral theory analyses reveal archaeal and bacterial communities are assembled differently in Hot Springs. Front. Bioeng. Biotech. 8:464. doi: 10.3389/fbioe.2020.00464, PMID: 32548097 PMC7271673

[ref45] Li H. Z. Yang Q. H. Li J. Gao H. Li P. Zhou H. Y. (2015). The impact of temperature on microbial diversity and AOA activity in the Tengchong geothermal field, China. Sci. Rep. 5:17056. doi: 10.1038/srep17056, PMID: 26608685 PMC4660298

[ref46] Lima-Mendez G. Faust K. Henry N. Decelle J. Colin S. Carcillo F.. (2015). Determinants of community structure in the global plankton interactome. Science 348:6237. doi: 10.1126/science.1262073, PMID: 25999517

[ref47] Liu K. Ding X. Wang J. (2020). Soil metabolome correlates with bacterial diversity and co-occurrence patterns in root-associated soils on the Tibetan plateau. Sci. Total Environ. 735:139572. doi: 10.1016/j.scitotenv.2020.139572, PMID: 32480142

[ref48] MacArthur R. (1955). Fluctuations of animal populations and a measure of community stability. Ecology 36, 533–536. doi: 10.2307/1929601

[ref49] Manish K. Pandit M. K. (2018). Phylogenetic diversity, structure and diversification patterns of endemic plants along the elevational gradient in the eastern Himalaya. Plant Ecol. Divers. 11, 501–513. doi: 10.1080/17550874.2018.1534147

[ref50] Meyer-Dombard D. R. Shock E. L. Amend J. P. (2005). Archaeal and bacterial communities in geochemically diverse hot springs of Yellowstone National Park, USA. Geobiology 3, 211–227. doi: 10.1111/j.1472-4669.2005.00052.x

[ref51] Miroshnichenko M. L. Bonch-Osmolovskaya E. A. (2006). Recent developments in the thermophilic microbiology of deep-sea hydrothermal vents. Extremophiles 10, 85–96. doi: 10.1007/s00792-005-0489-5, PMID: 16418793

[ref52] Nelson C. E. (2009). Phenology of high-elevation pelagic bacteria: the roles of meteorologic variability, catchment inputs and thermal stratification in structuring communities. ISME J. 3, 13–30. doi: 10.1038/ismej.2008.81, PMID: 18784755

[ref53] Neutel A. M. Heesterbeek J. A. P. de Ruiter P. C. (2002). Stability in real food webs: weak links in long loops. Science 296, 1120–1123. doi: 10.1126/science.1068326, PMID: 12004131

[ref54] Nevers M. B. Byappanahalli M. N. Nakatsu C. H. Kinzelman J. L. Phanikumar M. S. Shively D. A.. (2020). Interaction of bacterial communities and indicators of water quality in shoreline sand, sediment, and water of Lake Michigan. Water Res. 178:115671. doi: 10.1016/j.watres.2020.115671, PMID: 32380294

[ref55] Nielson K. G. Gill K. M. Springer A. E. Ledbetter J. D. Stevens L. E. Rood S. B. (2019). Springs ecosystems: vulnerable ecological islands where environmental conditions, life history traits, and human disturbance facilitate non-native plant invasions. Biol. Invasions 21, 2963–2981. doi: 10.1007/s10530-019-02025-6

[ref56] Nilsson K. A. McCann K. S. (2016). Interaction strength revisited—clarifying the role of energy flux for food web stability. Theor. Ecol. 9, 59–71. doi: 10.1007/s12080-015-0282-8

[ref57] Olesen J. M. Bascompte J. Dupont Y. J. Jordano P. (2007). The modularity of pollination networks. Proc. Natl. Acad. Sci. U. S. A. 104, 19891–19896. doi: 10.1073/pnas.0706375104, PMID: 18056808 PMC2148393

[ref58] Oliverio A. M. Power J. F. Washburne A. Cary S. C. Stott M. B. Fierer N. (2018). The ecology and diversity of microbial eukaryotes in geothermal springs. ISME J. 12, 1918–1928. doi: 10.1038/s41396-018-0104-2, PMID: 29662145 PMC6052046

[ref59] Olsen G. J. Woese C. R. Overbeek R. (1994). The winds of (evolutionary) change: breathing new life into microbiology. J. Bacteriol. 176, 1–6. doi: 10.1128/jb.176.1.1-6.1994, PMID: 8282683 PMC205007

[ref60] Putman L. I. Sabuda M. C. Brazelton W. J. Kubo M. D. Hoehler T. M. McCollom T. M.. (2021). Microbial communities in a Serpentinizing aquifer are assembled through strong concurrent dispersal limitation and selection. mSystems 6, e00300–e00321. doi: 10.1128/msystems.00300-21 34519519 PMC8547479

[ref61] Qian H. Zhang Y. J. Zhang J. Wang X. L. (2013). Latitudinal gradients in phylogenetic relatedness of angiosperm trees in North America. Glob. Ecol. Biogeogr. 22, 1183–1191. doi: 10.1111/geb.12069

[ref62] Qiu L. P. Zhang Q. Zhu H. S. Reich P. B. Banerjee S. van der Heijden M. G. A.. (2021). Erosion reduces soil microbial diversity, network complexity and multifunctionality. ISME J. 15, 2474–2489. doi: 10.1038/s41396-021-00913-1, PMID: 33712698 PMC8319411

[ref63] Quast C. Pruesse E. Yilmaz P. Gerken J. Schweer T. Yarza P.. (2013). The SILVA ribosomal RNA gene database project: improved data processing and web-based tools. Nucleic Acids Res. 41, D590–D596. doi: 10.1093/nar/gks1219, PMID: 23193283 PMC3531112

[ref64] Rath K. M. Rousk J. (2015). Salt effects on the soil microbial decomposer community and their role in organic carbon cycling: a review. Soil Biol. Biochem. 81, 108–123. doi: 10.1016/j.soilbio.2014.11.001

[ref65] Ren Z. Ma K. Jia X. Wang Q. Zhang C. Li X. (2022). Community assembly and co-occurrence patterns of microeukaryotes in Thermokarst Lakes of the Yellow River source area. Microorganisms 10:481. doi: 10.3390/microorganisms10020481, PMID: 35208934 PMC8877526

[ref66] Ren F. Yang X. X. Zhou H. K. Zhu W. Y. Zhang Z. H. Chen L. T.. (2016). Contrasting effects of nitrogen and phosphorus addition on soil respiration in an alpine grassland on the Qinghai-Tibetan plateau. Sci. Rep. 6:34786. doi: 10.1038/srep34786, PMID: 27721415 PMC5056390

[ref67] Ren Z. Zhang C. Li X. Ma K. Zhang Z. Feng K. X.. (2021). Bacterial communities present distinct co-occurrence networks in sediment and water of the Thermokarst Lakes in the Yellow River source area. Front. Microbiol. 12:716732. doi: 10.3389/fmicb.2021.716732, PMID: 34745028 PMC8569892

[ref68] Rillig M. C. Ryo M. Lehmann A. Aguilar-Trigueros C. A. Buchert S. Wulf A.. (2019). The role of multiple global change factors in driving soil functions and microbial biodiversity. Science 366, 886–890. doi: 10.1126/science.aay2832, PMID: 31727838 PMC6941939

[ref69] Sharp C. E. Brady A. L. Sharp G. H. Grasby A. E. Stott M. B. Dunfield P. F. (2014). Humboldt’s spa: microbial diversity is controlled by temperature in geothermal environments. ISME J. 8, 1166–1174. doi: 10.1038/ismej.2013.237, PMID: 24430481 PMC4030231

[ref70] Silvertown J. (2004). Plant coexistence and the niche. Trends Ecol. Evol. 19, 605–611. doi: 10.1016/j.tree.2004.09.003

[ref71] Sloan W. T. Lunn M. Woodcock S. Head I. M. Nee S. Curtis T. P. (2006). Quantifying the roles of immigration and chance in shaping prokaryote community structure. Environ. Microbiol. 8, 732–740. doi: 10.1111/j.1462-2920.2005.00956.x, PMID: 16584484

[ref72] Smith T. W. Lundholm J. T. (2010). Variation partitioning as a tool to distinguish between niche and neutral processes. Ecography 33, 648–655. doi: 10.1111/j.1600-0587.2009.06105.x

[ref73] Song Z. Q. Chen J. Q. Jiang H. C. Zhou E. M. Tang S. K. Zhi X. Y.. (2010). Diversity of crenarchaeota in terrestrial hot springs in Tengchong, China. Extremophiles 14, 287–296. doi: 10.1007/s00792-010-0307-6, PMID: 20373121

[ref74] Stegen J. C. Lin X. J. Fredrickson J. K. Chen X. Y. Kennedy D. W. Murray C. J.. (2013). Quantifying community assembly processes and identifying features that impose them. ISME J. 7, 2069–2079. doi: 10.1038/ismej.2013.93, PMID: 23739053 PMC3806266

[ref75] Stegen J. C. Lin X. Fredrickson J. K. Konopka A. E. (2015). Estimating and mapping ecological processes influencing microbial community assembly. Front. Microbiol. 6:370. doi: 10.3389/fmicb.2015.00370, PMID: 25983725 PMC4416444

[ref76] Thompson L. R. Sanders J. G. McDonald D. Amir A. Ladau J. Locey K. J.. (2017). A communal catalogue reveals Earth’s multiscale microbial diversity. Nature 551, 457–463. doi: 10.1038/nature24621, PMID: 29088705 PMC6192678

[ref77] Trivedi P. Leach J. E. Tringe S. G. Sa T. Singh B. K. (2020). Plant-microbiome interactions: from community assembly to plant health. Nat. Rev. Microbiol. 18, 607–621. doi: 10.1038/s41579-020-0412-1, PMID: 32788714

[ref78] Turner P. Mamo G. Karlsson E. N. (2007). Potential and utilization of thermophiles and thermostable enzymes in biorefining. Microb. Cell Factories 6:9. doi: 10.1186/1475-2859-6-9, PMID: 17359551 PMC1851020

[ref79] Walters K. E. Capocchi J. K. Albright M. B. N. Hao Z. Brodie E. L. Martiny J. B. H. (2022). Routes and rates of bacterial dispersal impact surface soil microbiome composition and functioning. ISME J. 16, 2295–2304. doi: 10.1038/s41396-022-01269-w, PMID: 35778440 PMC9477824

[ref80] Wang S. Hou W. G. Dong H. L. Jiang H. C. Huang L. Q. Wu G.. (2013). Control of temperature on microbial community structure in Hot Springs of the Tibetan plateau. PLoS One 8:e62901. doi: 10.1371/journal.pone.0062901, PMID: 23667538 PMC3647046

[ref81] Wang X. Q. Lu X. Li Z. Q. Cheng Q. Zhou Y. M. Lei M. (2021). Liming alters microbial community composition and its co-occurrence patterns in cd- and Pb-contaminated agricultural soil. Appl. Soil Ecol. 166:104064. doi: 10.1016/j.apsoil.2021.104064

[ref82] Wang X. Yin Y. Yu Z. Shen G. Cheng H. Tao S. (2023). Distinct distribution patterns of the abundant and rare bacteria in high plateau hot spring sediments. Sci. Total Environ. 863:160832. doi: 10.1016/j.scitotenv.2022.160832, PMID: 36521602

[ref83] Wangchuk P. Yeshi K. Ugyen K. Dorji J. Wangdi K. Samten S.. (2021). Water-based therapies of Bhutan: current practices and the recorded clinical evidence of Balneotherapy. Water 13:9. doi: 10.3390/w13010009, PMID: 39800344

[ref84] Webb C. O. (2000). Exploring the phylogenetic structure of ecological communities: an example for rain forest trees. Am. Nat. 156, 145–155. doi: 10.1086/303378, PMID: 10856198

[ref85] Webb C. O. Ackerly D. D. McPeek M. A. Donoghue M. J. (2002). Phylogenies and community ecology. Annu. Rev. Ecol. Syst. 33, 475–505. doi: 10.1146/annurev.ecolsys.33.010802.150448

[ref86] Wei G. S. Li M. C. Li F. G. Li H. Gao Z. (2016). Distinct distribution patterns of prokaryotes between sediment and water in the Yellow River estuary. Appl. Microbiol. Biotechnol. 100, 9683–9697. doi: 10.1007/s00253-016-7802-3, PMID: 27557722

[ref87] Weiher E. Clarke G. D. P. Keddy P. A. (1998). Community assembly rules, morphological dispersion, and the coexistence of plant species. Oikos 81, 309–322. doi: 10.2307/3547051

[ref88] Woese C. R. Kandler O. Wheelis M. L. (1990). Towards a natural system of organisms: proposal for the domains Archaea, Bacteria, and Eucarya. Proc. Natl. Acad. Sci. U. S. A. 87, 4576–4579. doi: 10.1073/pnas.87.12.4576, PMID: 2112744 PMC54159

[ref89] Wu W. X. Huang B. Q. (2019). Protist diversity and community assembly in surface sediments of the South China Sea. MicrobiologyOpen 8:e891. doi: 10.1002/mbo3.891, PMID: 31218846 PMC6813438

[ref90] Yang Q. Zhang P. Li X. D. Yang S. X. Chao X. Liu H. Q.. (2023). Distribution patterns and community assembly processes of eukaryotic microorganisms along an altitudinal gradient in the middle reaches of the Yarlung Zangbo River. Water Res. 239:120047. doi: 10.1016/j.watres.2023.120047, PMID: 37167854

[ref91] Ye X. F. Li Z. K. Luo X. Wang W. H. Li Y. K. Li R.. (2020). A predatory myxobacterium controls cucumber fusarium wilt by regulating the soil microbial community. Microbiome 8:49. doi: 10.1186/s40168-020-00824-x, PMID: 32252828 PMC7137222

[ref92] Zhang H. J. Liu Y. Yi L. Chao W. Zhang W. L. Wang L. F.. (2022). Pollution gradients shape the co-occurrence networks and interactions of sedimentary bacterial communities in Taihu Lake, a shallow eutrophic lake. J. Environ. Manag. 305:114380. doi: 10.1016/j.jenvman.2021.114380, PMID: 34995945

[ref93] Zhang P. Xiong J. Qiao N. Q. Luo S. Yang Q. Li X. D.. (2023). High variation in Protist diversity and community composition in surface sediment of Hot Springs in Himalayan Geothermal Belt, China. Microorganisms 11:674. doi: 10.3390/microorganisms11030674, PMID: 36985247 PMC10053680

[ref94] Zheng D. Zhao D. (2017). Characteristics of natural environment of the Tibetan plateau. Sci. Technol. Rev. 35, 13–22. doi: 10.3981/j.issn.1000-7857.2017.06.001

[ref95] Zhou J. Deng Y. Luo F. He Z. Tu Q. Zhi X. (2010). Functional molecular ecological networks. MBio 1, e00169–e00210. doi: 10.1128/mbio.00169-10 20941329 PMC2953006

[ref96] Zhou J. Ning D. (2017). Stochastic community assembly: does it matter in microbial ecology? Microbiol. Mol. Biol. Rev. 81, e00002–e00017. doi: 10.1128/mmbr.00002-17, PMID: 29021219 PMC5706748

