# Supplementary material for: The co-occurrence patterns and assembly mechanisms of microeukaryotic communities in geothermal ecosystems of the Qinghai-Tibet Plateau
Source: Front Microbiol. 2025 Feb 4;16:1513944. doi: 10.3389/fmicb.2025.1513944 (PMC11832674; doi:10.3389/fmicb.2025.1513944)
Supplement: Supplementary file 1 [file Data_Sheet_1.docx]

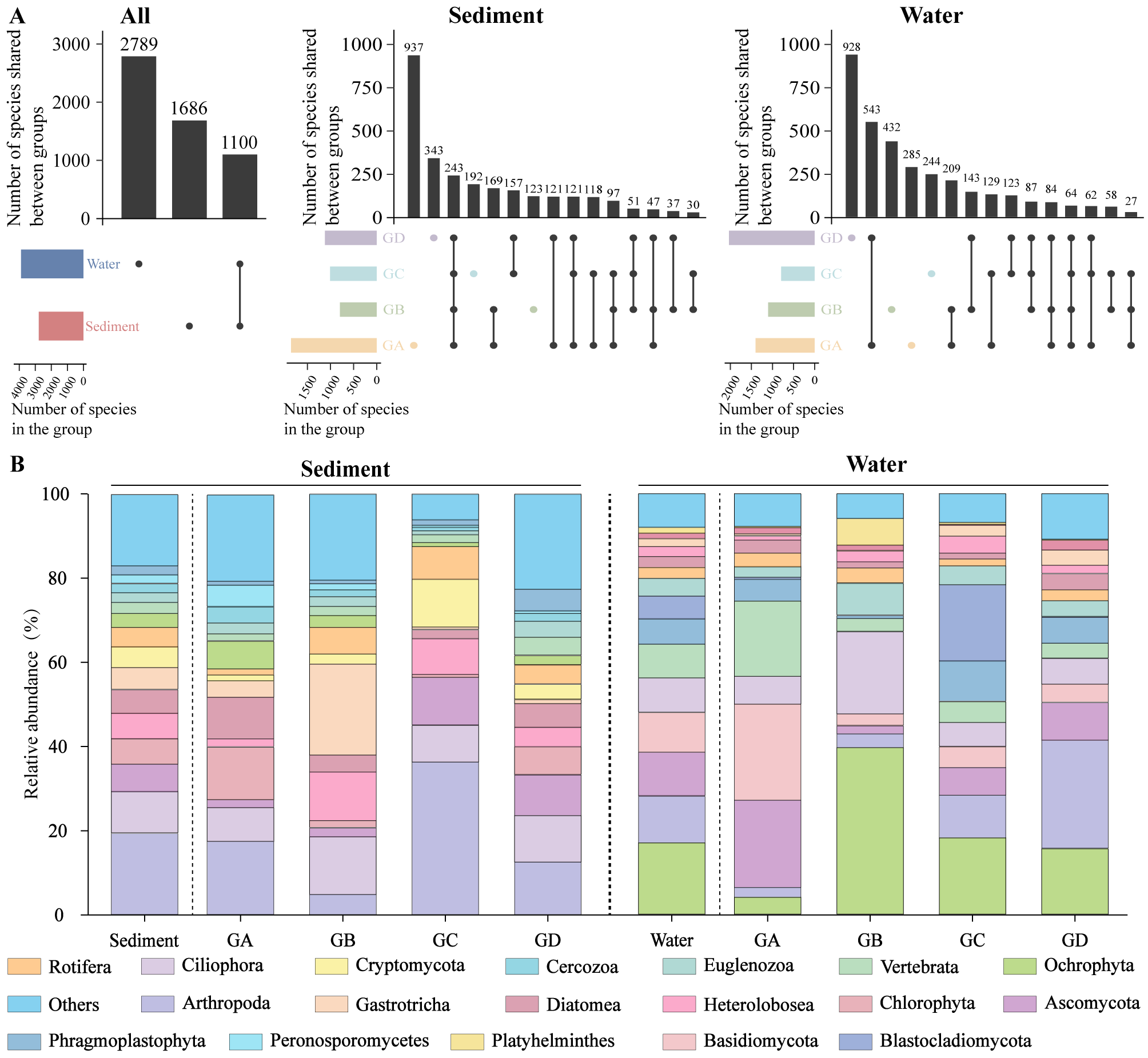


**Figure S1** Sediments and water microeukaryotic communities constitute. (A) Venn diagrams illustrating the total and shared as well as specific abundance of Amplicon Sequence Variants (ASVs) in sediments and water. (B) Temporal and spatial variations in the relative abundance of phylum-level ASVs in all. (GA: Group A, temperature ranging from 5 ℃ to 40 ℃; GB: Group B, temperature ranging from 40 ℃ to 50 ℃; GC: Group C, temperature ranging from 50 ℃ to 60 ℃, and GD: Group D, temperature exceeding 60 ℃.)


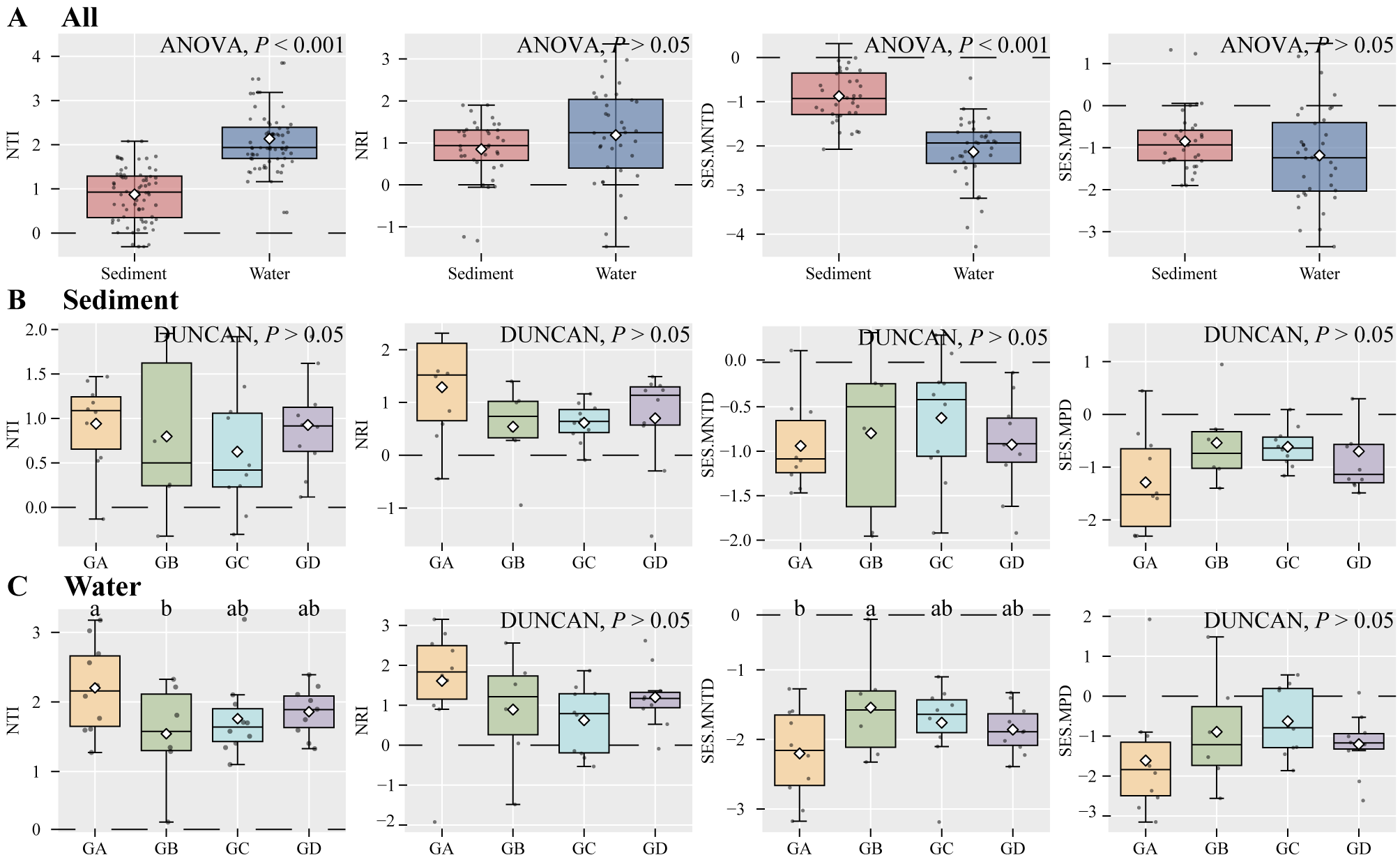


**Figure S2** Phylogenetic pattern. (A) Comparison in different habitats. (B) Comparison in sediments. (C) Comparison in water.


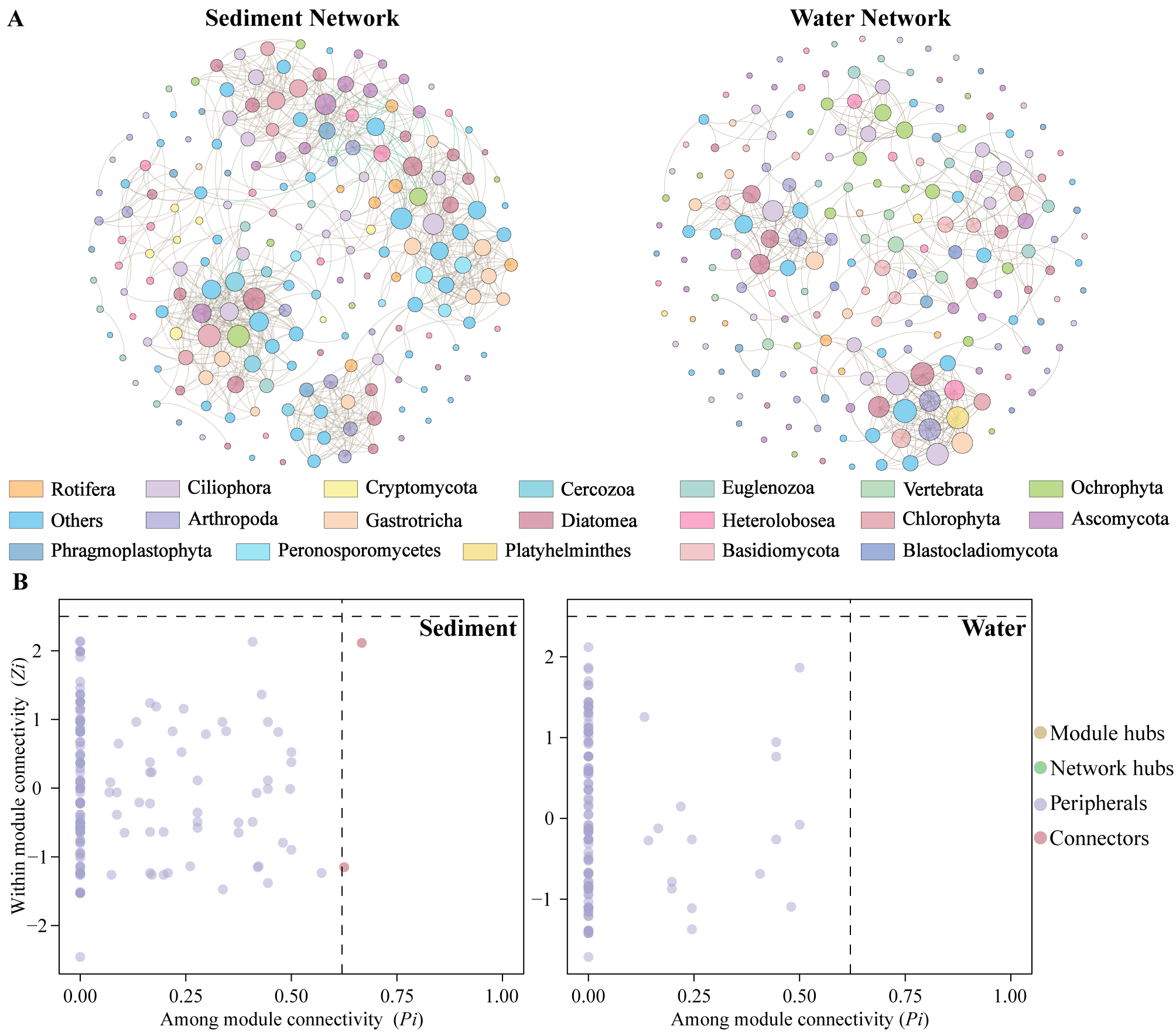


**Figure S3** Co-occurrence network analysis. (A) Co-occurrence networks under different habitats. (B) Keystone species analysis. Node size represents node degree; brown and green edges indicate positive and negative correlations between paired OTUs, respectively.


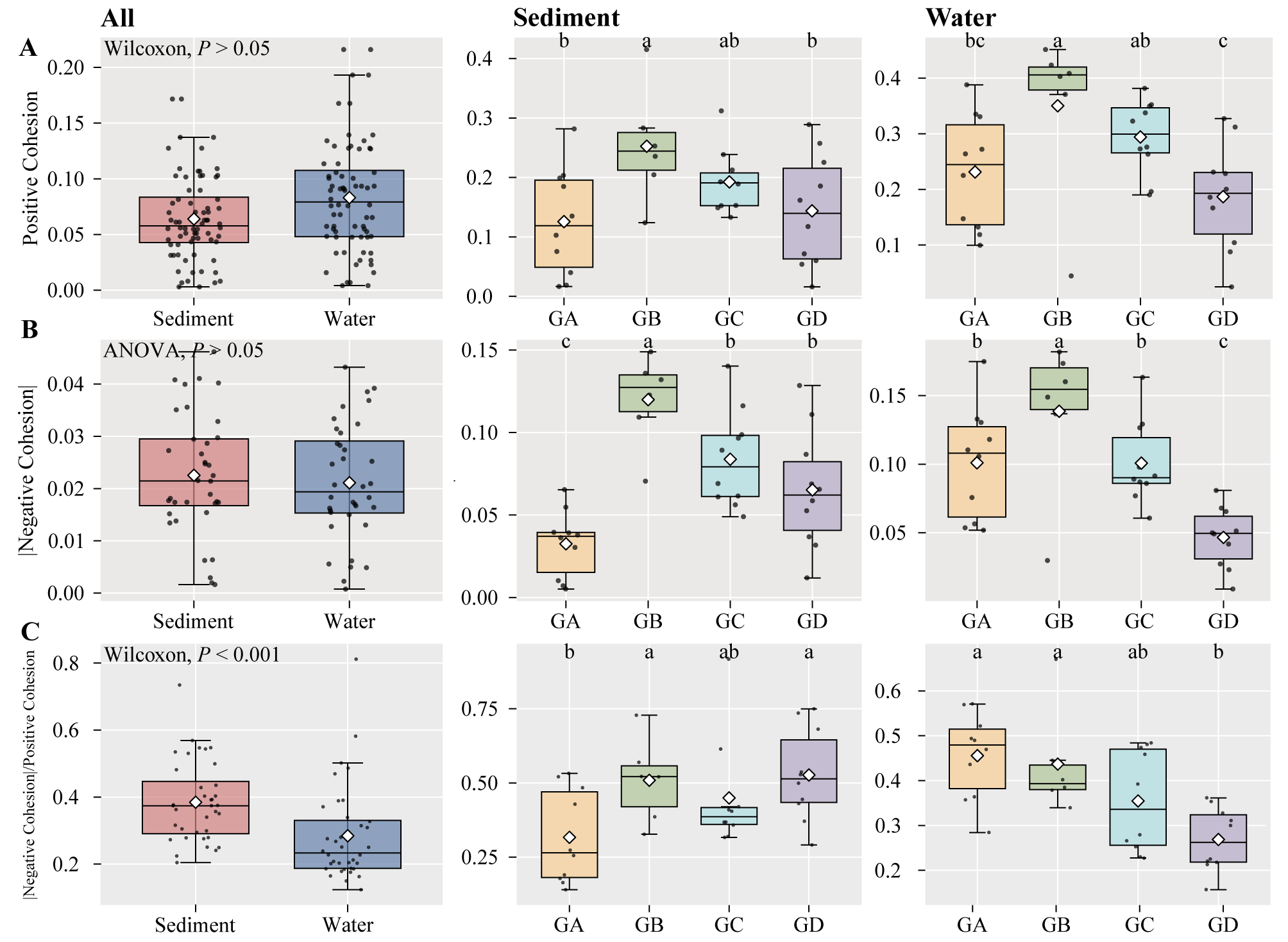


**Figure S4** Cohesion analysis. (A) Positive Cohesion Comparison. (B) Negative Cohesion Comparison. (C) Absolute value of negative cohesion to positive cohesion ratio. (**P* < 0.05; ***P* < 0.01; ****P* < 0.001).

**Table S1** Measured values of physical and chemical factors in the hot spring environment.

| **Sites** | **Sediment** | | | |  | **Water** | | | | |
| --- | --- | --- | --- | --- | --- | --- | --- | --- | --- | --- |
|  | pH | EC  (μs/cm) | ST  (℃) | Wg  (%) |  | pH | EC  (μs/cm) | WT  (℃) | TUR  (NTU) | NH_4_^+^-N  (mg/L) |
| GA1 | 6.65 | 1490 | 43.7 | 35.63 |  | 6.42 | 1594 | 30.0 | 0.53 | 0.12 |
| GA2 | 7.67 | 350 | 21.0 | 51.00 |  | 7.90 | 234 | 20.0 | 0.23 | 0.01 |
| GA3 | 7.23 | 850 | 18.0 | 50.40 |  | 7.64 | 515 | 17.0 | 0.60 | 0.00 |
| GA4 | 6.80 | 1260 | 17.5 | 38.83 |  | 6.62 | 1047 | 17.0 | 1.57 | 0.07 |
| GA5 | 6.55 | 2410 | 31.0 | 47.57 |  | 6.84 | 2205 | 31.0 | 0.20 | 0.26 |
| GA6 | 6.60 | 2790 | 20.5 | 43.83 |  | 6.36 | 1697 | 20.0 | 0.84 | 3.31 |
| GA7 | 6.70 | 3060 | 29.0 | 38.10 |  | 6.82 | 2824 | 28.0 | 21.93 | 1.89 |
| GA8 | 6.37 | 2470 | 37.0 | 39.30 |  | 6.69 | 704 | 36.0 | 2.33 | 0.36 |
| GA9 | 7.04 | 4360 | 21.0 | 43.03 |  | 6.57 | 5005 | 21.0 | 3.57 | 3.87 |
| GA10 | 8.82 | 280 | 23.0 | 38.47 |  | 8.59 | 380 | 22.0 | 0.58 | 0.03 |
| GB1 | 7.50 | 270 | 45.0 | 52.57 |  | 7.67 | 277 | 42.0 | 2.16 | 0.11 |
| GB2 | 7.61 | 1070 | 51.0 | 39.17 |  | 7.47 | 590 | 50.0 | 0.39 | 0.27 |
| GB3 | 6.82 | 2650 | 50.0 | 44.27 |  | 7.04 | 1750 | 50.0 | 31.83 | 3.87 |
| GB4 | 6.40 | 1150 | 49.0 | 41.37 |  | 8.53 | 495 | 49.0 | 0.34 | 2.11 |
| GB5 | 7.59 | 1650 | 48.0 | 39.03 |  | 7.61 | 1432 | 48.0 | 19.87 | 3.87 |
| GB6 | 7.20 | 800 | 45.0 | 28.53 |  | 6.74 | 1341 | 43.0 | 0.42 | 0.04 |
| GC1 | 7.05 | 810 | 55.0 | 39.47 |  | 7.03 | 384 | 55.0 | 5.76 | 0.70 |
| GC2 | 7.48 | 610 | 56.0 | 38.10 |  | 9.19 | 494 | 56.0 | 0.28 | 0.22 |
| GC3 | 6.83 | 4730 | 54.0 | 47.33 |  | 7.03 | 2268 | 53.0 | 1.24 | 0.15 |
| GC4 | 7.42 | 3680 | 59.0 | 43.27 |  | 6.56 | 5643 | 59.0 | 0.62 | 3.87 |
| GC5 | 7.79 | 1340 | 60.0 | 42.17 |  | 7.73 | 384 | 60.0 | 4.57 | 0.50 |
| GC6 | 7.72 | 1400 | 52.1 | 46.63 |  | 8.28 | 433 | 52.0 | 1.69 | 0.12 |
| GC7 | 8.14 | 950 | 54.0 | 39.70 |  | 8.09 | 848 | 54.0 | 0.32 | 0.38 |
| GC8 | 9.24 | 980 | 60.0 | 39.17 |  | 8.52 | 651 | 59.0 | 0.53 | 0.02 |
| GC9 | 8.29 | 630 | 56.0 | 39.20 |  | 7.08 | 904 | 54.0 | 1.66 | 0.51 |
| GC10 | 8.15 | 350 | 52.0 | 39.07 |  | 8.98 | 863 | 52.0 | 0.23 | 0.58 |
| GD1 | 7.31 | 2050 | 86.0 | 40.80 |  | 6.81 | 1854 | 84.0 | 0.99 | 0.27 |
| GD2 | 6.30 | 3040 | 92.0 | 45.23 |  | 8.26 | 1812 | 90.0 | 0.78 | 1.23 |
| GD3 | 6.90 | 3630 | 92.0 | 43.20 |  | 8.83 | 3140 | 91.0 | 2.19 | 3.87 |
| GD4 | 7.36 | 2090 | 90.0 | 39.80 |  | 8.53 | 2078 | 89.0 | 0.25 | 0.73 |
| GD5 | 6.85 | 2420 | 90.0 | 45.07 |  | 8.62 | 1140 | 90.0 | 3.10 | 0.09 |
| GD6 | 7.89 | 1370 | 43.0 | 40.20 |  | 7.53 | 1312 | 62.0 | 3.16 | 0.16 |
| GD7 | 9.37 | 4450 | 66.0 | 44.63 |  | 8.58 | 3835 | 66.0 | 11.42 | 2.17 |
| GD8 | 7.81 | 4100 | 66.0 | 45.93 |  | 7.10 | 2303 | 62.0 | 1.87 | 2.14 |
| GD9 | 9.05 | 1160 | 61.0 | 45.80 |  | 9.21 | 427 | 61.0 | 0.18 | 1.73 |
| GD10 | 10.18 | 2460 | 96.0 | 39.93 |  | 9.54 | 2934 | 95.0 | 1.29 | 1.63 |

**Table S2** Major topological properties of the empirical ecological networks of eukaryotic communities in water and sediment, and their associated random networks.

| Network properties | | All | | Sediment | | | | Water | | | |
| --- | --- | --- | --- | --- | --- | --- | --- | --- | --- | --- | --- |
|  |  | Sediment | Water | GA | GB | GC | GD | GA | GB | GC | GD |
| Empirical networks | Nodes | 200 | 200 | 178 | 155 | 157 | 168 | 166 | 128 | 139 | 163 |
|  | Edges | 858 | 378 | 1308 | 1091 | 1831 | 1783 | 1062 | 806 | 1014 | 987 |
|  | Positive | 809  (94.29%) | 378  (100%) | 1074  (82.11%) | 749  (68.65%) | 1443  (78.81%) | 1593  (89.34%) | 956  (90.02%) | 755  (93.67%) | 891  (87.87%) | 867  (87.84%) |
|  | Negative | 49  (5.71%) | 0  (0%) | 234  (17.89%) | 342  (31.35%) | 388  (21.19%) | 190  (10.66%) | 106  (9.98%) | 51  (6.33%) | 123  (12.13%) | 120  (12.16%) |
|  | avgD | 8.580 | 3.780 | 14.697 | 14.077 | 23.325 | 21.226 | 12.795 | 12.594 | 14.590 | 12.110 |
|  | Diameter | 11 | 10 | 6 | 10 | 7 | 8 | 6 | 8 | 6 | 6 |
|  | Density | 0.043 | 0.019 | 0.083 | 0.091 | 0.150 | 0.127 | 0.078 | 0.099 | 0.106 | 0.075 |
|  | avgCC | 0.624 | 0.642 | 0.545 | 0.724 | 0.673 | 0.656 | 0.555 | 0.786 | 0.568 | 0.555 |
|  | avgPL | 4.345 | 4.231 | 2.772 | 3.370 | 2.567 | 2.692 | 2.996 | 2.888 | 2.708 | 3.011 |
|  | Modularity | 0.794 | 0.794 | 0.864 | 1.160 | 0.837 | 0.759 | 0.577 | 0.634 | 0.698 | 0.770 |
|  | Closeness centrality | 0.244 | 0.249 | 0.367 | 0.497 | 0.409 | 0.377 | 0.337 | 0.634 | 0.376 | 0.336 |
|  | R^2^ of Power-law | 0.523 | 0.923 | 0.040 | 0.075 | 0.011 | 0.003 | 0.036 | 0.272 | 0.006 | 0.074 |
| Random networks | Modularity_r_  (SD) | 0.2718  (0.0128) | 0.4514  (0.0182) | 0.1935  (0.0093) | 0.197  (0.0097) | 0.1372  (0.0077) | 0.149  (0.008) | 0.210  (0.010) | 0.207  (0.012) | 0.189  (0.010) | 0.217  (0.010) |
|  | avgCC_r_  (SD) | 0.0429  (0.004) | 0.019  (0.006) | 0.083  (0.003) | 0.092  (0.004) | 0.149  (0.003) | 0.127  (0.003) | 0.077  (0.004) | 0.099  (0.005) | 0.105  (0.004) | 0.075  (0.004) |
|  | avgPL_r_  (SD) | 2.69  (0.0066) | 4.058  (0.057) | 2.188  (0.003) | 2.160  (0.004) | 1.876  (0.002) | 1.931  (0.002) | 2.266  (0.004) | 2.160  (0.005) | 2.085  (0.004) | 2.301  (0.004) |
|  | Small-world coefficient  (SD) | 8.559  (0.769) | 37.545  (17.229) | 4.911  (0.200) | 5.438  (0.225) | 3.289  (0.063) | 3.623  (0.078) | 7.200  (0.366) | 7.174  (0.342) | 4.521  (0.187) | 5.914  (0.312) |
